# Neural micro and macrostructural correlates of visual outcomes in children with unilateral cerebral palsy: A fixel-based study

**DOI:** 10.1162/IMAG.a.122

**Published:** 2025-08-26

**Authors:** Monica Crotti, Ahmed M. Radwan, Nofar Ben Itzhak, Lisa Mailleux, Lize Kleeren, Lisa Decraene, Hilde Feys, Els Ortibus

**Affiliations:** KU Leuven, Department of Development and Regeneration, Locomotor and Neurological Disorders group, Leuven, Belgium; KU Leuven, Child and Youth Institute, Leuven, Belgium; KU Leuven, Leuven Brain Institute, Department of Neurosciences, Leuven, Belgium; KU Leuven, Department of Imaging & Pathology, Translational MRI, Leuven, Belgium; KU Leuven, Department of Rehabilitation Sciences, Leuven, Belgium; Hasselt University, Rehabilitation Research Centre, Faculty of Rehabilitation Sciences, Diepenbeek, Belgium; University Hospitals Leuven, Department of Paediatric Neurology, Leuven, Belgium

**Keywords:** unilateral cerebral palsy, visual functions, white matter, fixel-based diffusion MRI, visual tracts

## Abstract

Children with unilateral cerebral palsy (uCP) present with brain damage, predominantly lateralized to one hemisphere, and white matter (WM) lesions, which are known to affect visual functions. However, the relation between WM tract damage and visual outcomes remains unclear. Additionally, no prior study comprehensively investigated hemispheric-specific differences in WM visual pathways between children with left- and right-sided uCP. Therefore, this exploratory study aims to investigate differences in micro- and macrostructural properties of the visual pathways between children with left- and right-sided uCP and their relation to visual outcomes, using fixel-based analysis of diffusion MRI (dMRI). dMRI data and visual assessments, including visual acuity and stereoacuity (i.e., geniculostriate functions), motor-free visual perception, visuomotor integration, and functional vision, were analysed in 36 children with uCP (aged 7–15, 9 males, 17 left-sided, 15 preterm). Apparent fiber density (AFD), fiber-bundle cross-section (FC), and combined fiber density and cross-section (FDC) were calculated for 17 WM tracts related to visual functions. Differences between children with left- and right-sided uCP were investigated using the Mann-Whitney *U*-test (*r*) on the AFD and one-way analysis of covariance (ANCOVA) (*η_p_^2^*) on the FC and FDC, with age and intracranial volume as covariates. Correlations between visual outcomes and WM properties of the visual tracts were studied using (semi-partial) Spearman Rank correlations (*r_s_*). Children with left-sided uCP showed significantly lower fixel metrics in the right superior longitudinal fasciculus, inferior fronto-occipital fasciculus, and optic radiation. Children with right-sided uCP had lower AFD, FC, and FDC in the left superior longitudinal fasciculus only. Reduced geniculostriate visual functions and more impairments in functional vision were associated with lower fiber density (AFD), reduction in bundle size (FC), and their combination (FDC) of several WM tracts. Lower performance on motor-free visual perception and visuomotor integration showed more associations with lower fiber density (AFD). While the primary analyses were exploratory and uncorrected for multiple comparison, false discovery rate (FDR) correction was additionally performed for transparency: several differences in FC and FDC between children with left- and right-sided uCP, and correlations between AFD and visual function, remained significant and are reported in the Supplementary Materials. In conclusion, our exploratory study highlights that fixel-based analysis can provide further insights into hemispheric differences in the visual system and the complex relations between visual functions and brain damage in children with uCP. Based on our results, future studies could refine regression models to target key WM tracts linked to visual outcomes, identifying potential biomarkers to predict visual impairments and enable early tailored support in children with uCP.

## 1 Introduction

Vision plays a crucial role in children’s daily activities by guiding social interaction, learning, and adaptation to the environment (Fabbro et al., 2020). The visual system entails a complex neural network where information from the retina travels through the optic radiation (OR) to the primary visual cortex in the occipital lobe (Swienton & Thomas, 2014). Visual information is then processed through two interacting systems, the dorsal stream, from the visual cortex to the parietal and frontal lobes, responsible for spatial perception and exploration, and the ventral stream, from the visual cortex to the temporal lobes, involved in object recognition (Cloutman, 2013; Sheth & Young, 2016). White matter (WM) tracts play a crucial role in visual processing by connecting different brain areas. Previous studies showed that in the dorsal stream, the superior longitudinal fasciculus (SLF) facilitates spatial awareness and visual attention, and the inferior fronto-occipital fasciculus (IFOF) is involved in visuomotor integration. Additionally, the inferior longitudinal fasciculus (ILF) part of the ventral stream, contributes to object recognition (Bauer & Merabet, 2024; Nakajima et al., 2020; Ortibus et al., 2012). Recent studies also highlighted the involvement of the vertical occipital fasciculus (VOF), connecting the dorsal and ventral areas of the occipital lobe (Bauer & Merabet, 2024; Jitsuishi et al., 2020; Oishi et al., 2018; Takemura et al., 2016) in encoding object properties (e.g., form, identity, and color) and mapping spatial information. The posterior section of the corpus callosum (CC) also plays a key role in visual functions by enabling interhemispheric communication, which is crucial for integrating visual information across both hemispheres (Berlucchi, 2014; Crotti, Ben Itzhak, et al., 2024; Martín-Signes et al., 2024). Depending on the location and the severity of damage, early brain lesions can affect the integrity of the WM connections of the visual system, resulting in different visual impairments (Dutton et al., 2006). Although low-level visual processing involves both hemispheres (Braddick & Atkinson, 2011), there has been considerable debate regarding hemispheric differences in visual functions processing between the left and right hemispheres, with the latter considered specialized for visual-perceptual functions (Hellige et al., 2010). Previous findings suggest that the left hemisphere primarily supports functions such as language and fine motor skills through intra-hemispheric connectivity, whereas the right hemisphere, which exhibits stronger cross-hemispheric connections, is more involved in visuospatial and attentional processing (Hellige et al., 2010). Therefore, lesion lateralization should be considered when studying the relation between brain damage and visual functions. This consideration is especially relevant for children with unilateral cerebral palsy (uCP), a motor neurodevelopmental disability caused by pre- and/or perinatal brain lesions predominantly lateralized to one hemisphere (Graham et al., 2016), in which visual impairment is a well-recognized comorbidity (Crotti, Ortibus, Mailleux, et al., 2024; Duke et al., 2022; Ego et al., 2015). Previous findings showed that children with uCP present with impaired visual acuity, stereoacuity, and visual-perceptual functions which can negatively affect their motor function and quality of life (Berelowitz & Franzsen, 2021; Crotti, Ortibus, Ben Itzhak, et al., 2024; Crotti, Ortibus, Mailleux, et al., 2024). Additionally, previous studies showed that children with left-sided uCP perform worse on visual-perceptual tests than children with neurotypical development or right-sided uCP, supporting the hypothesis of hemispheric differences in visual functions. Nevertheless, the authors did not report any information on brain damage (Berelowitz & Franzsen, 2021; Burtner et al., 2006).

To evaluate the relation between visual impairment and brain damage, qualitative (Himmelmann et al., 2017) and semiquantitative assessments (Fiori et al., 2014) based on structural magnetic resonance imaging (sMRI) have been used in children with cerebral palsy (CP), showing that WM lesions, particularly periventricular leukomalacia, are most frequently associated with visual impairment (Crotti, Genoe, et al., 2024; Petri & Tinelli, 2023). Nevertheless, previous findings also reported that 8 to 11% of children with CP do not show any abnormalities on sMRI, suggesting that this methodology might not be sensitive enough to detect subtle WM micro- and macrostructural damage (Crotti, Genoe, et al., 2024). For instance, our previous findings showed no difference in sMRI between children with left and right-sided uCP on the WM lobar and the total score of the semi-quantitative scale developed by Fiori et al. (2014) (Crotti, Ortibus, Mailleux, et al., 2024). Additionally, damaged brain tissue may still contribute to visual processing through reorganization within the affected cortex. For instance, damage to one hemisphere or a specific region can activate alternative pathways or neighboring areas to take over visual processing functions typically controlled by the compromised regions (Araneda et al., 2022; Grasso et al., 2020; Guzzetta, 2010). Therefore, we could hypothesize that these neuroplastic adaptations may involve the development of new connections to bypass the lesion or the functional differentiation of nearby tissue, changes that may be undetectable on sMRI (Guzzetta et al., 2010). Although sMRI is the standard method used in clinical practice, advanced imaging modalities, such as diffusion-weighted MRI (dMRI), can provide a deeper understanding of hemispheric differences in the visual pathways and the neural correlates of visual impairment in children with uCP. To investigate this relation, previous dMRI studies used the diffusion tensor imaging (DTI) model (Mori et al., 1999), a voxel-based analysis method quantifying the diffusion properties of water molecules within each three-dimensional pixel. Results showed that in children with spastic CP, lower fractional anisotropy in specific WM visual pathways was related to impairments in different visual functions (Ceschin et al., 2015; Galli et al., 2018; Rai et al., 2013). Furthermore, recent studies found that WM damage, calculated on the voxel-based metrics of the optic radiations, correlates with lower performance in visuospatial function (Wilson et al., 1987), particularly in children with right-sided lesions (i.e., left-sided uCP) (Araneda et al., 2022) and that fractional anisotropy and mean diffusivity of the optic radiations differ between lesioned and non-lesioned hemispheres in children with uCP due to stroke (Maiani et al., 2024). Despite the importance of these findings, only two studies explored potential differences in WM properties between children with left- and right-sided uCP, focusing only on the microstructural properties of the optic radiations (Araneda et al., 2022; Maiani et al., 2024). Additionally, DTI has been criticized for being inaccurate in detecting WM changes in regions containing crossing fibers (i.e., two or more fiber bundles with different orientations within a voxel) such as the SLF, the posterior thalamic radiations, and CC (Farquharson et al., 2013; Jeurissen et al., 2013). This limitation arises because DTI measures are based on a single-tensor model that assumes a single dominant fiber orientation within each voxel, making it unable to resolve complex fiber architecture in regions with crossing fibers. As a result, DTI may underestimate or fail to detect micro- and macrostructural differences in such regions (Farquharson et al., 2013). To overcome this limitation, constrained spherical deconvolution (CSD) has been developed, allowing the quantification of the signal at the level of fixels (i.e., fibers with a single orientation within a voxel) (Dhollander et al., 2021; Raffelt et al., 2017). This results in the calculation of metrics, namely apparent fiber density (AFD), fiber-bundle cross-section (FC), and fiber density and cross-section (FDC) which provide information on micro- and macrostructural properties of WM tracts (Raffelt et al., 2017). Whereas AFD quantifies the density of the fibers in a specific orientation within a voxel (microstructural), FC measures the cross-sectional area of a fiber bundle (macrostructural). Lastly, FDC, combines AFD and FC, providing a more comprehensive assessment of the micro- and macrostructural properties of WM.

Previous studies comparing DTI and CSD metrics on early WM development showed that childhood is marked by significant increases in the size of macroscopic fiber bundles (FC), modest changes in axonal density (AFD), and relatively minor changes in DTI metrics (Dimond et al., 2020). Furthermore, research on children with disabilities highlighted the advantages of using CSD over DTI for investigating WM organization in younger populations (Hyde et al., 2018). However, only one previous study used CSD analysis to investigate the relation between WM tracts of the visual system and visual outcomes, specifically in infants born very preterm (VPT; ≤32 weeks gestational age) (Chandwani et al., 2022). Results showed that the FDC of the left posterior thalamic radiation, ILF, and IFOF were significantly associated with visual attention scores of the Preverbal Visual Assessment (Pueyo et al., 2014), while no associations were found with visuomotor coordination or visual processing (Chandwani et al., 2022). To the best of our knowledge, no previous research used fixel-based metrics to comprehensively explore micro- and macrostructural differences in the WM properties of the visual system between children with left- and right-sided uCP, and their relation to visual outcomes. Hence, we conducted a comprehensive exploration of (1) differences in micro- and macrostructural properties of the visual pathways between children with left- and right-sided uCP and (2) the relation between these structural properties and visual outcomes in the whole group of children with uCP.

We hypothesized that WM properties of visual pathways would differ between children with left- and right-sided uCP, based on previous findings on the OR in children with stroke. Additionally, based on previous literature, we expected that lower fixel metrics in specific WM tracts would be associated with impairments in distinct visual outcomes in children with uCP.

## 2 Methods

### 2.1 Participants and procedure

Children with spastic uCP, aged between 7 and 15, were recruited via the CP care program of the University Hospitals Leuven (Belgium). This research is part of a cross-sectional study involving visual, sensorimotor, and MRI assessments. A full overview of the inclusion and exclusion criteria can be found in prior publications of our group (Crotti, Ben Itzhak, et al., 2024; Crotti, Ortibus, Ben Itzhak, et al., 2024; Crotti, Ortibus, Mailleux, et al., 2024; Decraene et al., 2023; Mailleux et al., 2024). Visual assessments and MRI were conducted by three trained researchers (M.C., L.D., and L.K) either in a single day or divided across two half-days based on the family’s preferences. The presence of comorbidities (epilepsy, autism spectrum disorder, attention-deficit/hyperactivity disorder, cognitive and hearing impairments) was retrieved through questionnaires filled by caregivers and through medical records. Gestational age, birth weight, side of uCP (i.e., the side with stronger upper limb motor impairment), and the level of manual ability classified according to the Manual Ability Classification System (MACS) (Eliasson et al., 2006) were retrieved from medical records. Lesion timing was classified according to the MRI classification system (MRICS) (Himmelmann et al., 2017). Consent and assent to participate were provided by parents and children older than 12 years, respectively. This study was approved by the UZ/KU Leuven Ethical Committee (S62906).

### 2.2 Measures

#### 2.2.1 Visual assessments

To assess visual functions and functional vision, we performed standardized and age-appropriate tests conducted with both eyes open and best-corrected vision, selected based on previous studies in CP (Ben Itzhak et al., 2021; Berelowitz & Franzsen, 2021; Burtner et al., 2006; Dufresne et al., 2014; Duke et al., 2022; Ego et al., 2015; Fazzi et al., 2012). Visual assessments were scored in a double-blind manner by a trained researcher (M.C.) and two pediatric physiotherapy trainees, all of whom were blind to the imaging data. A full description of the assessments and the cut-offs used to score visual impairments can be found in Crotti, Ortibus, Mailleux, et al. (2024).

The Freiburg Visual Acuity Test (FrACT) software (Bach, 1996) was used to investigate *visual acuity (VA).* Results were scored as a continuous variable in LogMAR (logarithm of the minimum angle of resolution = −log10[decimal acuity] (Holladay, 2004)), where higher values indicate lower levels of visual acuity. The FrACT test shows good test-retest variability and high testability already in preschool children (Farassat et al., 2024). The fly and the circle subtests of Titmus Stereo Fly (Stereo Optical Corporation, 2024) were administered to study *binocular stereoacuity*. Results were scored as ordinal numbers (0-9) with higher values indicating better stereoacuity. Previous studies showed that the Titmus Stereo Fly has good sensitivity to detect impairments in stereoacuity (Cruz et al., 2016; Moganeswari et al., 2015). The visual discrimination, spatial relationships, form constancy, visual figure-ground, and visual closure subtests of the Test of Visual Perceptual Skills, Fourth Edition (TVPS-4) (Martin, 2017) were used to investigate *motor-free visual-perceptual skills* and the visuomotor integration subtest (VMI) of the Beery-Buktenica Test of Visual-Motor Integration, Sixth Edition (Beery-VMI) (Beery et al., 2010) to study *visuomotor integration*. After converting the scaled scores of the TVPS-4 subtests and the standard scores of the VMI into age-equivalent scores based on the manuals, results were transformed into standardized *z*-scores (mean = 0, SD = 1), where higher scores indicate better visual-perceptual functions. The TVPS-4 can be administered from 5 years of age and showed acceptable internal-consistency reliability and evidence for content validity, construct validity, and criterion validity (Brown & Peres, 2018; Martin, 2017) and the Beery-VMI, which can be administered from the age of 2 years (Beery et al., 2010), presents with acceptable to excellent internal consistency, good reliability, and excellent inter-scorer reliability (McCane, 2006).

The Flemish cerebral visual impairment questionnaire (FCVIQ), a 46-item binary-response tool filled by parents, was used to assess *functional vision*, namely the use of vision in daily life (Colenbrander, 2005; Ortibus et al., 2011). Responses were calculated as a total score [0–46] given by the sum of the ‘yes’ items (1: the child presents the characteristic described in the item; 0: characteristic not present), where a higher score reflects a higher level of functional vision impairment (Ortibus et al., 2011). The FCVIQ has a good predictive value, good sensitivity, good internal consistency, and moderate specificity (Gorrie et al., 2019; Ortibus et al., 2011).

#### 2.2.2 MRI

##### 2.2.2.1 MRI acquisition

MRI was acquired with a 3.0 Tesla scanner (Achieva dStream, Philips Medical Systems, Best, The Netherlands) with a 32-channel head coil. SMRI included the acquisition of T1-weighted images (TE/TR/TI 4.2/9.1/760.3 *ms*, voxel size: 0.9 × 0.9 × 0.9 *mm*³), T2-weighted images (TE/TR/TI 280/3000/548 *ms*, voxel size: 1 × 1 × 1 *mm*^3^), and T2 fluid attenuation inversion recovery images (FLAIR) (TE/TR/TI 283/4800/1650 *ms*, voxel size: 1 × 1 × 1 *mm*^3^). Multi-shell, multi-tissue constrained spherical deconvolution (MSMT-CSD) dMRI was performed using a 2D spin-echo Echo Planar imaging technique with the following parameters: TR/TE 3765/93 *ms*, voxel size: 2.2 x 2.2 x 2.2 *mm³*, matrix: 108 x 106 x 62, anterior–posterior phase encoding direction, 3 b0 images, 50 diffusion directions with a b-value of 1,000 *s/mm²* and 74 diffusion directions with a b-value of 2,500 *s/mm²*), and reversed-phase dMRI (1 b0 image with same parameters in posterior-anterior phase encoding direction). To prevent motion artifacts, children were explicitly instructed to remain as still as possible during the scan, and this was reinforced through a familiarization protocol prior to the MRI session (Verly et al., 2019). Additionally, after the acquisition of each MRI sequence, the data were immediately available for quality assessment. In cases where excessive motion artifacts were detected for the T1, T2 and FLAIR images, the affected sequence was re-acquired to ensure optimal data quality.

##### 2.2.2.2 Image processing

*SMRI* data (T1 and FLAIR) was used to assess lesion location and extent, using a semi-quantitative scale validated for children with CP (Fiori et al., 2014). A detailed description of the methodology and results is available in our prior publication (Crotti, Ben Itzhak, et al., 2024).

**Fig. 1.**
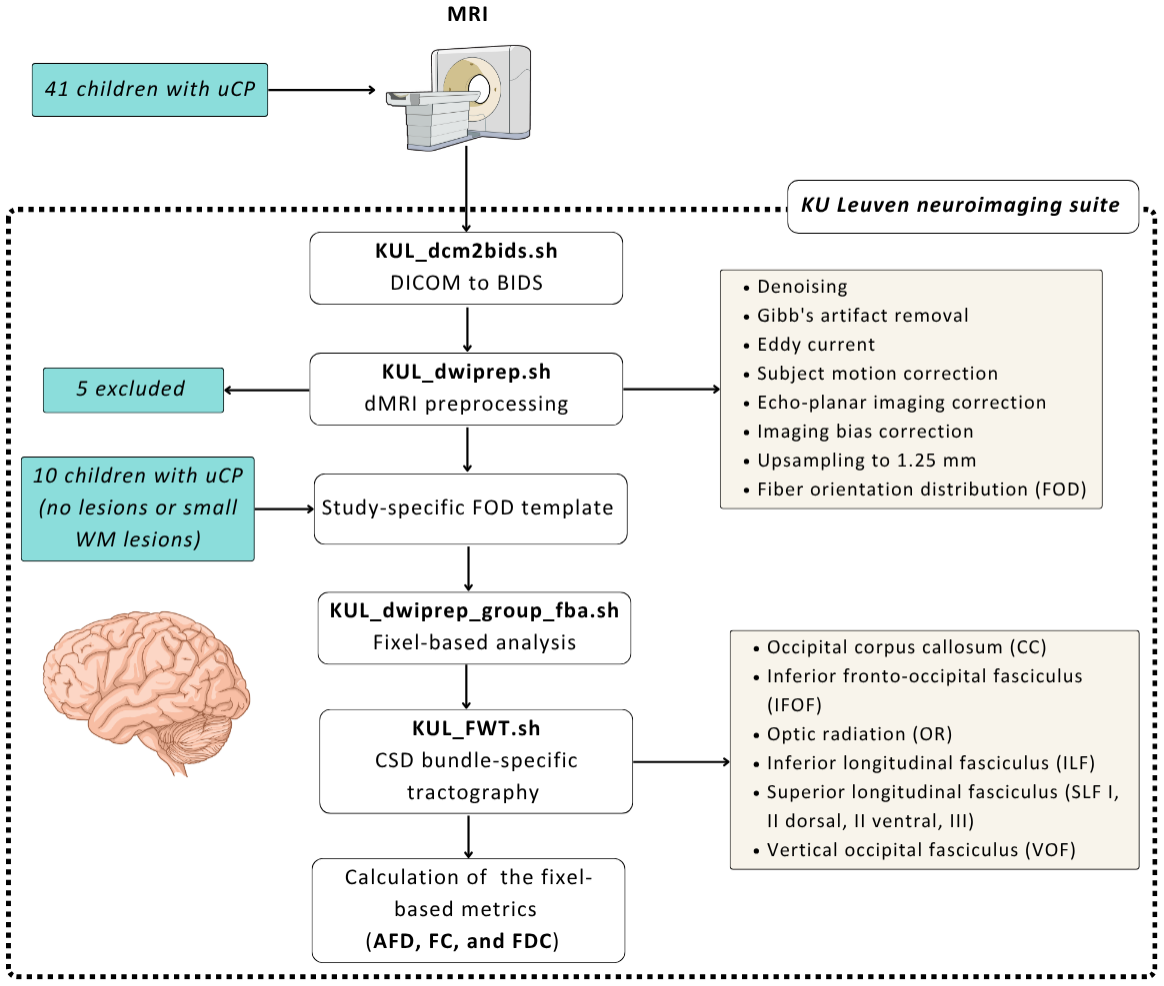
Schematic representation of the diffusion MRI preprocessing and analysis workflow. uCP, unilateral cerebral palsy, BIDS, brain imaging data structure; dMRI, diffusion magnetic resonance imaging; FOD, fiber orientation distribution; KUL_FWT, KU Leuven fun with tracts; CSD, constrained spherical deconvolution; AFD, Apparent fiber density; FC, fiber-bundle cross-section; FDC, combined fiber density and cross-section.

*DMRI* preprocessing was performed and reviewed in consultation with a neuroradiologist (A.M.R.), who was blinded to the visual assessments, using custom-developed scripts developed by the neuroradiology department of KU and UZ Leuven (Fig. 1). First, sMRI and dMRI images were converted to the brain imaging data structure (BIDS) format using the KU Leuven neuroimaging suite (Radwan & Sunaert, 2018/2024) and *dcm2bids* (Bedetti et al., 2022). Secondly, dMRI preprocessing was performed using the script *KUL_dwiprep.sh* (Radwan & Sunaert, 2018/2024) which relies on FSL (v6.0) (Jenkinson et al., 2012), ANTs (v2.3.0) (Avants et al., 2011), and MRtrix3 (v3.0.3) (Tournier et al., 2019) for denoising using Marchenko-Pastur-Principal Component Analysis (MP-PCA) (Veraart et al., 2016), Gibb’s artifact removal (Kellner et al., 2016), Eddy current artifact, and subject motion correction using FSL’s eddy (Andersson & Sotiropoulos, 2016), echo-planar imaging correction using a FSL’s topup (Andersson et al., 2003), and imaging bias correction (Tustison et al., 2010). This was followed by upsampling of the diffusion images (1.25 *mm*) and the computation of fiber orientation distribution (FOD). Lastly, the diffusion signal was clustered based on the degree of restriction and anisotropy of three different tissues, namely white matter, grey matter, and cerebrospinal fluid (Jeurissen et al., 2014).

**Fig. 2.**
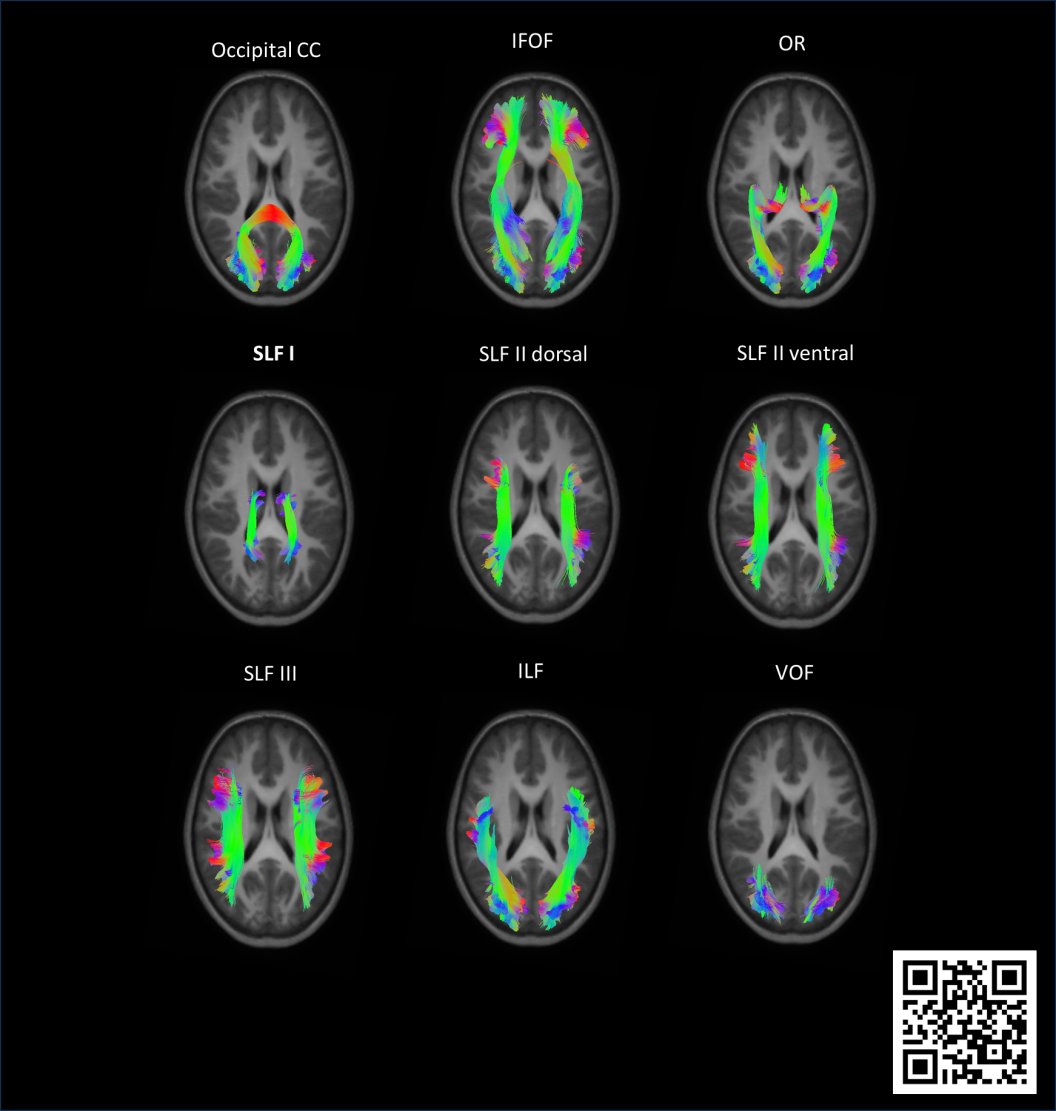
Representative reconstruction from the population template in axial view of the occipital corpus callosum (CC), the right and left inferior fronto-occipital fasciculus (IFOF), optic radiation (OR), four subdivisions of the superior longitudinal fasciculus (SLF: I, II dorsal, II ventral, III), inferior longitudinal fasciculus (ILF), and the vertical occipital fasciculus (VOF) (Radwan et al., 2021). The 3D reconstructions of the visual tracts in sagittal view can be visualized by scanning the QRcode through the app https://www.schol-ar.io/download/ (Ard et al., 2022). The RGB color scheme is applied to indicate the orientation of fiber tracts: red for left-right, green for anterior-posterior, and blue for superior-inferior. In the 3D reconstructions, white occurs when the left and right bundles are superimposed during rotation due to the bundles’ semitransparency.

To investigate the micro- and macrostructural properties of the WM visual tracts, a fixel-based analysis was performed using an automated BASH implementation of the MRtrix3 (Tournier et al., 2019) workflow (*KUL_dwiprep_group_fba.sh*) (Radwan & Sunaert, 2018/2024). A study-specific multi-contrast FOD template was generated using the data of 10 included children with uCP, reporting no visible lesions or small predominant WM lesions according to the MRICS (mean age: 10 years and 9 months; left-sided uCP = 3; males = 3; preterm = 4; PVL = 8, normal MRI = 2), which were flipped across the x-axis, resulting in 20 images used for the generation of a symmetrical population template. This approach minimizes the influence of uCP lesion laterality on the resulting population template. To exclude lesioned voxels from the template creation, lesion masks were manually generated using ITK-snap (v.3.8.0) on the T1- and T2 FLAIR-weighted images, which encoded the lesion as Not a Number (NaNs) and a cost function masking was used to minimize inaccuracies due to the presence of focal pathology (Andersen et al., 2010). Following the generation of the template, the FOD maps of each participant were warped to the FOD population template with *mrregister* and the resulting subject fixels were reoriented to the corresponding template fixels using *fixelreorient* (Tournier et al., 2019). The Fun With Tracts automated pipeline (Radwan et al., 2021) was used to perform fully automated probabilistic CSD bundle-specific tractography using iFOD2 (Second-order Integration over Fiber Orientation Distributions) (Tournier et al., 2010) for the following tracts of interest: occipital CC, the right and left inferior fronto-occipital fasciculus (IFOF), optic radiation (OR), inferior longitudinal fasciculus (ILF), four subdivisions of the superior longitudinal fasciculus (SLF: I, II dorsal, II ventral, III), and vertical occipital fasciculus (VOF) (Fig. 2). For a full description of the inclusion/exclusion volumes of interest (VOIs), see supplementary table 2 in Radwan et al. (2021). With the FWT pipeline, we first created the VOIs based on the neuroanatomical literature using the parcellation output of FreeSurfer recon-all (Fischl, 2012; *FreeSurferWiki, 2020*) and MultiScale Brain Parcellator (Tourbier et al., 2019).

Secondly, preprocessed dMRI and VOIs were used to generate bundle-specific tractograms for the population template (Radwan et al., 2021). The resulting tractograms were used to define template bundle-specific fixel masks, which were then used to sample each subject’s fixel metrics in template space. During registration, NaN masks were applied to minimise the influence of lesions, and all registered images were visually inspected for accuracy.

For each participant, the resulting bundle-specific fixel maps were used to calculate the fixel-based metrics (i.e., AFD, FC, and FDC). FC was logarithmically transformed to simplify data interpretation: with a value of 0 corresponding to the same FC as the template, values above 0 indicating an increase in FC, and values below 0 indicating a decrease. In the following sections, FC refers to the log-transformed FC. For each participant, the fixel-based metrics were calculated as the average values across the entirety of each tract, where lower values indicate a reduction in the size of fiber bundles (FC), loss of fiber density (AFD) and/or a combination of both (FDC) (Raffelt et al., 2017). Additionally, the intracranial brain volume was estimated using the brain masks obtained during the preprocessing (Pannek et al., 2018; Raffelt et al., 2017).

### 2.3 Statistical analyses

Frequencies were calculated for descriptive characteristics and visual impairments. Normality of the data was evaluated using the Shapiro-Wilk test. Quantitative variables were described using the mean and standard deviation (SD) or the median and interquartile range (IQR) based on the data distribution, and parametric or non-parametric statistics were performed accordingly. Based on the literature (Chandwani et al., 2022; Pannek et al., 2018; Peters et al., 2012), to identify potential confounders related to WM properties of the visual tracts in children with uCP, pairwise Spearman’s Rank correlations, adjusted for false discovery rate (FDR *p*-value ≤ 0.05) (Benjamini & Hochberg, 1995), were performed between the AFD, FC, and FDC of the visual tracts and gestational age and age, which were not normally distributed. A similar analysis was performed between visual outcomes and gestational age (Chandwani et al., 2022). The relation between visual outcomes and age was not investigated, since participants were aged between 7 and 15 years, and, according to the literature, visual acuity and stereoacuity are fully developed by the third year of life (Braddick & Atkinson, 2011). Furthermore, the results of the TVPS-4 and VMI were already corrected for the age-equivalent scores reported in the manuals (Beery et al., 2010; Martin, 2017). Additionally, we assessed whether males and females showed differences in brain damage and visual outcomes using Mann-Whitney *U*-tests with FDR correction. Following the fixel-based framework described by Raffelt et al. (2017), we used intracranial volume as a covariate for analyses including FC and FDC to remove the global effects of brain scaling resulting from the registration to the study template. A correction for intracranial volume was not applied for AFD, since its calculation does not involve brain scaling (Raffelt et al., 2017; Smith et al., 2019). Since we do not hypothesize that differences in head size (i.e., intracranial volume) impact visual outcomes in children with uCP, a correction for intracranial volume was not applied to the results of the visual assessments. Correlation coefficients (*r_s_*) were interpreted as no or negligible (<0.30), low (0.30–0.49), moderate (0.50–0.69), high (0.70–0.89), or very high (≥0.90) (Mukaka, 2012). To compare baseline differences between children with left- and right-sided uCP, we conducted Mann-Whitney *U*-tests for continuous variables (age, intracranial volume, gestational age) and Chi-square tests for the categorical variable (sex). Lastly, to exclude potential confounders, we evaluated the extent of the lesion for the right and left hemispheres using the semi-quantitative scale of Fiori et al. (2014). Although we found that three children with right-sided uCP had a more extensive lesion in the hemisphere ipsilateral to their motor impairments (i.e., right), when comparing between groups the extent of the lesion on the lobar and total WM scores (Fiori et al., 2014), there were no significant differences between children with right and left-side uCP (Crotti, Ben Itzhak, et al., 2024). Data were analyzed using R (version 4.3.2; R Core Team, 2021).

#### 2.3.1 Differences in white matter properties of the visual tracts between children with left- and right-sided uCP

Based on the data distribution, a Mann-Whitey *U* test for AFD and one-way analysis of covariance (ANCOVA) for FC and FDC was performed to investigate differences in WM properties of the visual tracts between children with left- and right-sided uCP. For the Mann-Whitey *U* test analysis, effect sizes were calculated using correlation coefficients (*r*) (Tomczak & Tomczak, 2014) and interpreted as small (<0.3), medium (0.3–0.49), or large (≥0.5) (Mukaka, 2012). A one-way ANCOVA was conducted to examine the differences between groups (i.e., children with left- and right-sided uCP) on the FC and FDC of the visual tracts while controlling for age and ICV. The assumption of normality of residuals was checked with the Shapiro-Wilk test, homoscedasticity with the Levene’s test, and the homogeneity of the regression slopes with the interaction between the group and each covariate (group × age and group × ICV). If this interaction was not statistically significant, interaction terms were removed from the ANCOVA analysis (Leppink, 2018). Effect sizes were calculated using partial *η* squared (*η_p_^2^*) and interpreted as small (0.01–0.06), medium (0.06–0.14), or large (>0.14) (Hinkle et al., 2003). Two-sided *p*-values ≤0.05 were considered statistically significant. Given the exploratory nature of the study, the primary analyses were conducted without correction for multiple comparisons. However, to ensure transparency, FDR was performed post hoc, and the results are available in the Supplementary Materials.

#### 2.3.2 The relation between white matter properties of the visual tracts and visual outcomes

To study the univariate associations between WM properties of the visual tracts and visual outcomes in children with uCP, we performed (1) pairwise semi-partial Spearman’s Rank correlations between the results of the visual assessments and the FDC and FC of the visual tracts, with corrections for age and intracranial volume applied on the FDC and FC, and (2) Spearman rank correlations without covariate correction between the visual outcomes and the AFD of the visual tracts. Correlation coefficients (*r_s_*) were interpreted according to Mukaka (2012). Correction for multiple comparisons was not applied to the primary analysis because of the exploratory nature of this study (Bender & Lange, 2001; Rothman, 1990). However, to ensure transparency, FDR correction was performed post hoc, and the results are available in the Supplementary Materials.

## 3 Results

### 3.1 Participants

Fifty children with uCP were recruited for this study. Nine children were excluded due to contraindications to MRI assessments, and five children due to failure of the motion and distortion correction during the preprocessing of the dMRI images. Particularly, one subject was excluded due to a large structural lesion that caused FreeSurfer reconstruction failure. Four additional subjects were excluded due to significant artefacts detected during preprocessing. Exclusion was based on a combination of quality control metrics (e.g., motion, Signal-to-Noise Ratio, Contrast-to-Noise Ratio, outlier counts) and the persistence of artefacts after applying multiple correction methods (including dwifslpreproc, FSL’s eddy, and SHARD). In all excluded cases, data quality was deemed insufficient for reliable analysis. Therefore, 36 children with uCP (mean age 11 years 7 months, SD 2 years 10 months, 19 males, 17 left-sided uCP, 15 preterm) were included in the statistical analysis. A detailed overview of missing data is presented in Supplementary Figure S1. Descriptive characteristics and comorbidities of our sample are presented in Table 1, and results of the visual assessments and the fixel metrics are presented in Supplementary Tables S1 and S2, respectively. A detailed description of lesion locations in this cohort is available in the Supplementary Figure S2.

**Table 1.** Clinical characteristics of children with unilateral cerebral palsy.

General characteristics		n	(%)
Mean age (SD), years:months	11:07		
	2:10		
Sex	Male	19	53
	Female	17	47
Handedness	Right-handed	17	47
	Left-handed	19	53
Side of cerebral palsy	Right-sided	19	53
	Left-sided	17	47
Magnetic Resonance Imaging Classification System category^a^ (Himmelmann et al., 2017)	A	2	6
	B	24	67
	C	7	19
	D	1	3
	E	2	6
Manual Ability Classification System level^a^ (Eliasson et al., 2006)	I	19	53
	II	13	36
	III	4	11
Gestational age^a^^,^^b^	Mean (SD), weeks:days	36:05 (3:6)	
	Term	20	56
	Preterm	10	28
	Very preterm	5	14
	Unknown^d^	1	3
Birth weight^a^^,^^c^	Mean (SD), grams	2,898.23 (969.95)	
	Normal	26	72
	Low	9	25
	Unknown^d^	1	3
Intracranial brain volume (ICV)	Median (IQR), cm^3^	1,348.845 (102.696)	

^a^Comorbidities	Category	n	(%)
Epilepsy	No epilepsy	26	72
	Epilepsy	10	28
Autism spectrum disorder (ASD)	No ASD	27	75
	ASD	9	25
Attention deficit hyperactivity disorder (ADHD)	No ADHD	33	92
	ADHD	3	8
Cognitive impairments	No	33	92
	Yes	3	8
Hearing impairments	No	36	100
	Yes	0	0

Percentages are calculated out of the total sample of children with unilateral CP (*N *= 36).

a Retrieved from medical records.

b Gestational age refers to completed weeks of pregnancy: term, ≥37 to <42 weeks; preterm, ≥32 to <37 weeks; very preterm, <32 weeks (Tucker & McGuire, 2004).

c Birthweight: normal birthweight, ≥2,500 g; low birthweight, <2,500 g (World Health Organization., 2014).

d Unknown reflects no reported data or missing data, which exists because of the retrospective data retrieval.

CP, cerebral palsy; SD, standard deviation; IQR, interquartile range. A, maldevelopments; B, predominant white matter injury; C, predominant grey matter injury; D, miscellaneous; E, normal.

### 3.2 Covariates selection

After applying FDR correction, no significant correlations were found between gestational age and WM properties of the visual tracts (AFD, FC, FDC; Supplementary Table S3) or the visual outcomes (Supplementary Table S4). Significant correlations were found between age and the FDC (*r_s_* = 0.379–0.455) and FC (*r_s_* = 0.370–0.610) of the visual tracts, but not with the AFD of the visual tracts (Supplementary Table S5). Additionally, after applying FDR correction, no significant differences were found between children with left- and right-sided uCP for age, gestational age, ICV, and sex (Supplementary Table S6), and between males and females on the visual outcomes and WM properties of the visual tracts (Supplementary Table S7). Therefore, only age and intracranial volume were standardized and used as covariates for FC and FDC.

### 3.3 Difference in white matter properties of the visual tracts between children with left- and right-sided uCP

**Fig. 3.**
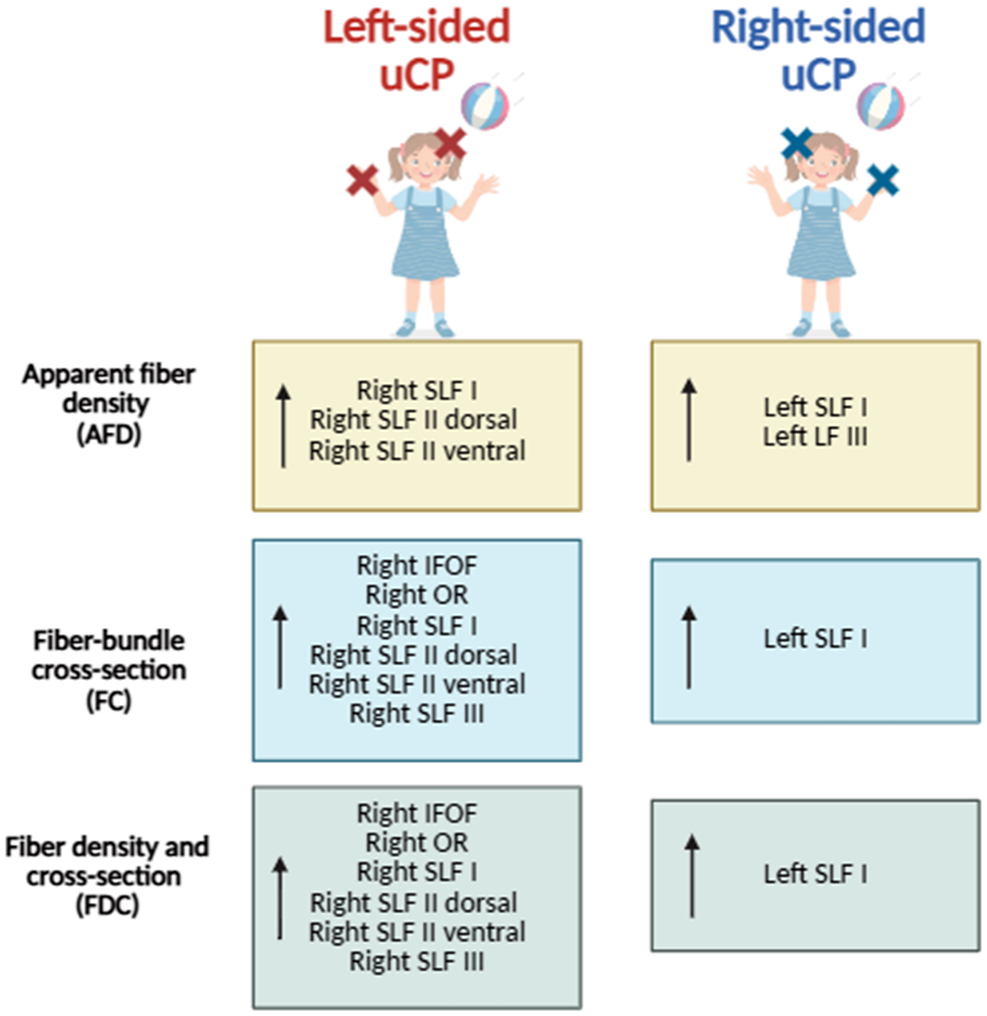
Graphical representation summarizing differences between children with left-sided and right-sided unilateral cerebral palsy (uCP) on the fixel-metrics, namely (in yellow) apparent fiber density (AFD), (in blue) fiber-bundle cross-section (FC), and (in green) fiber density and cross-section (FDC). The upward arrow (↑) denotes white matter tracts with greater damage. uCP, unilateral cerebral palsy; SLF, superior longitudinal fasciculus; IFOF, inferior fronto-occipital fasciculus; OR, optic radiation.

A full overview of the Mann–Whitney *U* tests for AFD and ANCOVA results for FC and FDC with the relative effect sizes (*r, η_p_^2^*) is presented in Table 2 and graphically summarized in Figure 3. Significant interaction effects were only found between group and age for the right VOF for the FC (*p* = 0.016; *η_p_^2^* = 0.177) and FDC (*p* = 0.042; *η_p_^2^* = 0.131) (Supplementary Table S8), showing a medium to large effect size, indicating that for the right VOF, age influences the presence of micro- and macrostructural differences between children with left- and right-sided uCP. The results on the main effect of group showed that, with medium to large effect sizes, children with left-sided uCP have significantly lower AFD (*p* = 0.021–0.018; *r* = 0.383–0.393), FC (*p* = 0.012–0.001; *η_p_^2^* = 0.182–0.299), and FDC (*p* = 0.002–<0.001; *η_p_^2^* = 0.256–0.323) in different branches of the right SLF compared to children with right-sided uCP, and children with right-sided uCP showed significantly lower AFD (*p* = 0.028–0.007; *r* = 0.367–0.441), FC (*p* = 0.012; *η_p_^2^* = 0.183), and FDC (*p* = 0.01; *η_p_^2^* = 0.192) in different branches of the left SLF. Additionally, children with left-sided uCP showed significantly lower FDC and FC in the right IFOF (FC: *p* = 0.009; *η_p_^2^* = 0.193; FDC: *p* = 0.029; *η_p_^2^* = 0.140) and right OR (FC: *p* = 0.011; *η_p_^2^* = 0.187; FDC: *p* = 0.018; *η_p_^2^* = 0.162) compared to children with right-sided uCP. None of the AFD results remained significant following FDR correction, whereas several group differences in FC and FDC did; these are detailed in Supplementary Table S9.

**Table 2.** Differences in fixel metrics of the visual tracts between children with left- and right-sided uCP.

	Mann-Whitney *U* test on AFD				ANCOVA corrected for age and ICV on FC				ANCOVA corrected for age and ICV on FDC			
Visual tracts	Median [IQR] of children with left uCP	Median [IQR] of children with right uCP	***p***	Effect size (***r***)	Mean (SD) of children with left uCP	Mean (SD) of children with right uCP	Main effect group		Mean (SD) of children with left uCP	Mean (SD) of children with right uCP	Main effect group	
							***p***	Effect size (***η_p_^2^***)			***p***	Effect size (***η_p_^2^***)
Occipital CC	0.55 [0.06]	0.56 [0.06]	0.471	0.124	-0.04 (0.22)	-0.05 (0.26)	0.380	0.024	0.52 (0.15)	0.54 (0.19)	0.244	0.042
Left IFOF	0.47 [0.03]	0.45 [0.06]	0.379	0.151	0.01 (0.18)	-0.09 (0.24)	0.334	0.029	0.49 (0.10)	0.42 (0.15)	0.242	0.042
Right IFOF	0.47 [0.06]	0.48 [0.03]	0.146	0.246	**-0.06 (0.19)**	***0.02 (0.15)***	**0.009**	**0.193**	**0.45 (0.12)**	***0.50 (0.09)***	**0.029**	**0.140**
Left ILF	0.46 [0.04]	0.45 [0.05]	0.379	0.151	0.08 (0.24)	-0.01 (0.28)	0.517	0.013	0.52 (0.13)	0.45 (0.17)	0.401	0.022
Right ILF	0.45 [0.06]	0.46 [0.04]	0.271	0.187	0.00 (0.28)	0.08 (0.22)	0.073	0.097	0.46 (0.16)	0.50 (0.11)	0.074	0.096
Left OR	0.46 [0.04]	0.46 [0.04]	0.510	0.114	0.00 (0.23)	-0.08 (0.26)	0.629	0.007	0.48 (0.12)	0.43 (0.16)	0.546	0.012
Right OR	0.48 [0.08]	0.49 [0.04]	0.165	0.235	**-0.10 (0.24)**	***-0.01 (0.18)***	**0.011**	**0.187**	**0.44 (0.14)**	***0.50 (0.11)***	**0.018**	**0.162**
Left SLF I	***0.46 [0.03]***	**0.44 [0.07]**	**0.010**	**0.425**	***0.11 (0.24)***	**-0.16 (0.38)**	**0.012**	**0.183**	***0.53 (0.13)***	**0.38 (0.19)**	**0.010**	**0.192**
Right SLF I	**0.41 [0.07]**	***0.46 [0.03]***	**0.018**	**0.393**	**-0.14 (0.27)**	***0.06 (0.23)***	**0.012**	**0.182**	**0.38 (0.13)**	***0.50 (0.13)***	**0.001**	**0.285**
Left SLF IId	0.42 [0.04]	0.40 [0.11]	0.107	0.272	-0.01 (0.24)	-0.17 (0.34)	0.149	0.064	0.45 (0.13)	0.34 (0.18)	0.074	0.097
Right SLF IId	**0.39 [0.12]**	***0.42 [0.02]***	**0.021**	**0.383**	**-0.20 (0.26)**	***0.00 (0.22)***	**0.002**	**0.260**	**0.32 (0.13)**	***0.44 (0.13)***	**<0.001**	**0.323**
Left SLF III	***0.40 [0.03]***	**0.38 [0.13]**	**0.007**	**0.441**	-0.02 (0.24)	-0.17 (0.3)	0.200	0.051	0.42 (0.12)	0.32 (0.16)	0.057	0.109
Right SLF III	0.39 [0.10]	0.40 [0.02]	0.071	0.304	**-0.18 (0.22)**	***-0.01 (0.2)***	**0.001**	**0.299**	**0.32 (0.12)**	***0.42 (0.11)***	**0.001**	**0.285**
Left SLF IIv	***0.40 [0.04]***	**0.38 [0.11]**	**0.028**	**0.367**	0.00 (0.22)	-0.16 (0.31)	0.172	0.057	0.42 (0.11)	0.33 (0.15)	0.087	0.089
Right SLF IIv	0.37 [0.10]	0.39 [0.02]	0.244	0.198	**-0.17 (0.23)**	***0.00 (0.2)***	**0.003**	**0.247**	**0.32 (0.12)**	***0.41 (0.12)***	**0.002**	**0.256**
Left VOF	0.42 [0.05]	0.42 [0.05]	0.490	0.119	0.11 (0.26)	0.01 (0.31)	0.495	0.015	0.49 (0.16)	0.43 (0.16)	0.396	0.023
Right VOF	0.46 [0.05]	0.47 [0.06]	0.315	0.172	0.02 (0.24)	0.06 (0.23)	0.151	0.065	0.48 (0.14)	0.51 (0.14)	0.118	0.077

Significant results are shown in bold: *p ≤ 0.05, **p ≤ 0.01. The median or mean of the group showing the higher properties of the visual tracts is highlighted in italics.

AFD, apparent fiber density; FC, fiber-bundle cross-section; FDC, fiber density and cross-section; ICV, intracranial volume; CC, corpus callosum; IFOF, inferior fronto-occipital fasciculus; ILF, inferior longitudinal fasciculus; OR, optic radiation; SLF, superior longitudinal fasciculus; d, dorsal; v, ventral; VOF, vertical occipital fasciculus; IQR, interquartile range; uCP, unilateral cerebral palsy; *p*; *p*-value; *r*, effect size calculated as correlation coefficients (*r* *=* *𝑍*/ √*𝑛*, where *Z* is the standardized value for the U-value and *n* the total number of observations) (Tomczak & Tomczak, 2014) and interpreted as small (<0.3), medium (0.3–0.5), or large (≥0.5) (Mukaka, 2012); *η_p_^2^*, effect size calculated using partial *η* squared and interpreted as small (0.01–0.06), medium (0.06–0.14), or large (>0.14) (Hinkle et al., 2003).

### 3.4 The relation between visual outcomes and white matter properties of the visual tracts

**Fig. 4.**
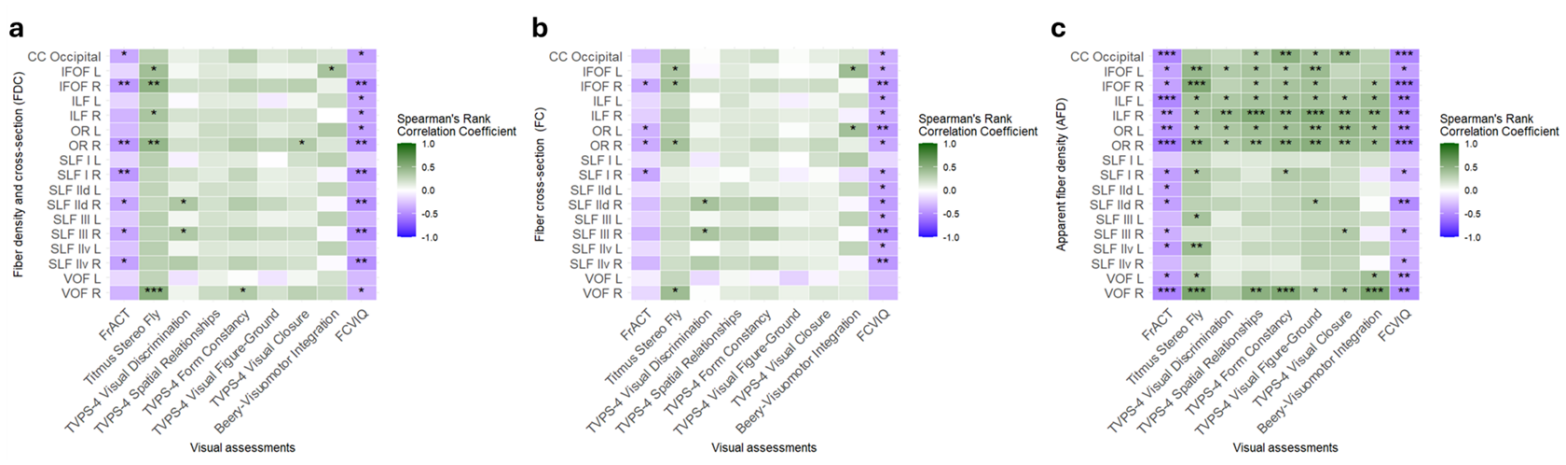
Partial Spearman’s rank correlation matrix showing the significant correlations between the visual assessments and the fixel-metrics, namely (a) fiber density and cross-section (FDC), (b) fiber-bundle cross-section (FC), and (c) apparent fiber density (AFD). CC, corpus callosum; L, left; R, right; IFOF, inferior fronto-occipital fasciculus; ILF, inferior longitudinal fasciculus; OR, optic radiation; SLF, superior longitudinal fasciculus; d, dorsal; v, ventral; VOF, vertical occipital fasciculus; FrACT, Freiburg Visual Acuity Test; TVPS-4, Test of Visual Perceptual Skills, Fourth Edition; Beery, Beery-Buktenica Test of Visual-Motor Integration, Sixth Edition; FCVIQ, Flemish cerebral visual impairment questionnaire. *r_s_*, Spearman correlations interpreted as no or negligible correlation (<0.30), low (0.30–0.49), moderate (0.50–0.69), high (0.70–0.89), or very high (≥0.90) (Mukaka, 2012); *p*, *p*-value.

A full overview of the Spearman’s Rank correlations between the visual outcomes and the FDC, FC, and AFD of the visual tracts is presented in Tables 3, 4, and 5, respectively, and graphically in Figure 4. In summary, we found low to moderate correlations between the visual outcomes and the micro- and macrostructural properties of several visual pathways. In the present section, when not specified, correlations were found with low effect sizes (*r_s_* = 0.30–0.49). Lower *visual acuity* was associated with lower FDC of the occipital CC, the right IFOF, OR, SLF I, SLF IId, SLF IIv, and SLF III (*r_s_* = -0.371 to -0.445), and lower FC of the right IFOF, SLF I and bilateral OR (*r_s_* = -0.335 to -0.377). Low to moderate correlations were found between visual acuity and lower AFD of almost all the visual pathways (*r_sv_*= -0.345 to -0.557). Lower *stereoacuity* was correlated with lower FDC of the bilateral IFOF, the right ILF, OR (*r_s_* = 0.367-0.485), and with moderate effect sizes with lower FDC of the right VOF (*r_s_* = 0.511), with lower FC of the right OR, VOF, and bilateral IFOF (*r_s_* = 0.359–0.420), and lower AFD of the bilateral ILF, OR, right SLF I and left SLF IIv, SLF III, IFOF, and VOF (*r_s_* = 0.336–0.461), and with moderate effect sizes with the right IFOF and VOF (*r_s_* = 0.543–0.571). Regarding *motor-free visual-perceptual functions*, lower scores on the TVPS-4 subtest *visual discrimination* were associated with lower FDC of the right SFL IId and SLF III (*r_s_* = 0.341–0.350), lower FC of the right SLF IId and SLF III (*r_s_* = 0.381–0.362), and lower AFD of the bilateral ILF and OR, and left IFOF (*r_s_* = 0.329–0.443). Lower scores on the *form constancy* and *visual closure* subtests were associated with lower FDC of the right VOF (*r_s_* = 0.334) and right OR (*r_s_* = 0.330), respectively, and AFD of several WM tracts (*r_s_* = 0.329–0.488) with the *form constancy* subtest showing moderate effect sizes with the occipital CC and right VOF (*r_s_* = 0.503–0.549). Lower scores on the *spatial relationship* and *visual figure-ground* subtests were associated only with lower AFD of several WM tracts (*r_s_* = 0.335–497). More specifically, the *spatial relationship* subtest showed moderate correlations with the AFD of the right ILF (*r_s_* = 0.541), and the *visual figure-ground* subtest with the right ILF and OR (*r_s_* = 0.508–0.540). Lower scores on the *VMI* were associated with lower FDC of the left IFOF only (*r_s_* = 0.380), lower FC of the left OR and IFOF (*r_s_* = 0.396–0.417), and lower AFD of the bilateral ILF, OR, left VOF, and right IFOF (*r_s_* = 0.358–0.463), with a moderate effect size with the right VOF (*r_s_* = 0.575). Lastly, higher impairments in *functional vision (FCVIQ)* were associated with lower FC (*r_s_* = -0.338 to -0.483) of all the visual tracts except the VOF, and lower FDC (*r_s_* = -0.334 to -0.499) and AFD of all visual tracts (*r_s_* = -0.357 to -0.499), with associations with the AFD reporting moderate correlations with the occipital CC, right OR, IFOF, VOF, and left ILF (*r_s_* = -0.502 to -0.558). None of the correlations with FC or FDC remained significant after FDR correction, whereas several AFD correlations did; these are reported in Supplementary Table S10.

**Table 3.** Semi-partial Spearman Rank’s correlations corrected for age and ICV between the results of the visual assessments and the FDC of the visual tracts.

	**Fiber density and cross-section (FDC)**																	
Visual assessments		Occipital CC	Left IFOF	Right IFOF	Left ILF	Right ILF	Left OR	Right OR	Left SLF I	Right SLF I	Left SLF IId	**Right SLF IId**	Left SLF III	Right SLF III	Left SLF IIv	Right SLF IIv	Left VOF	Right VOF
FraCT	*r_s_*	-0.371^*^	-0.175	-0.438^**^	-0.185	-0.321	-0.295	-0.445^**^	-0.187	-0.443^**^	-0.229	-0.381^*^	-0.227	-0.380^*^	-0.258	-0.402^*^	-0.164	-0.322
	*p*	0.026	0.308	0.008	0.279	0.056	0.081	0.007	0.276	0.007	0.179	0.022	0.183	0.022	0.129	0.015	0.338	0.056
Titmus Stereo Fly	*r_s_*	0.307	0.402^*^	0.485^**^	0.296	0.367^*^	0.293	0.456^**^	0.295	0.256	0.267	0.246	0.296	0.281	0.299	0.289	0.267	0.511^***^
	*p*	0.069	0.015	0.003	0.08	0.028	0.083	0.005	0.081	0.132	0.116	0.148	0.08	0.097	0.077	0.087	0.116	0.001
TVPS-4 Visual Discrimination	*r_s_*	0.099	0.045	0.203	-0.022	0.175	0.099	0.193	-0.063	0.189	0.095	0.350^*^	0.057	0.341^*^	0.056	0.327	-0.122	0.069
	*p*	0.564	0.797	0.236	0.897	0.307	0.565	0.258	0.715	0.269	0.583	0.036	0.741	0.042	0.745	0.052	0.479	0.687
TVPS-4 Spatial Relationships	*r_s_*	0.161	0.142	0.197	0.124	0.197	0.22	0.212	0.138	0.126	0.197	0.19	0.189	0.217	0.150	0.194	0.081	0.225
	*p*	0.348	0.408	0.250	0.473	0.248	0.198	0.215	0.424	0.465	0.249	0.266	0.269	0.204	0.383	0.257	0.638	0.187
TVPS-4 Form Constancy	*r_s_*	0.310	0.134	0.290	0.119	0.226	0.223	0.32	0.124	0.258	0.18	0.321	0.158	0.291	0.131	0.287	0.013	0.334^*^
	*p*	0.066	0.436	0.086	0.491	0.185	0.191	0.057	0.473	0.129	0.294	0.056	0.357	0.085	0.447	0.09	0.942	0.047
TVPS-4 Visual Figure-Ground	*r_s_*	0.155	0.058	0.265	-0.086	0.193	0.118	0.268	-0.007	0.102	0.101	0.242	0.072	0.243	0.128	0.222	-0.111	0.186
	*p*	0.366	0.738	0.118	0.616	0.259	0.493	0.115	0.966	0.555	0.558	0.154	0.677	0.153	0.458	0.193	0.519	0.278
TVPS-4 Visual Closure	*r_s_*	0.213	0.061	0.298	0.057	0.198	0.168	0.330^*^	0.195	0.128	0.126	0.177	0.101	0.23	0.144	0.186	0.049	0.289
	*p*	0.213	0.723	0.078	0.742	0.246	0.326	0.050	0.256	0.458	0.465	0.301	0.559	0.177	0.403	0.276	0.776	0.088
Beery- Visuomotor integration	*r_s_*	0.153	0.380^*^	0.087	0.216	-0.001	0.33	0.152	0.282	-0.047	0.256	-0.028	0.273	-0.03	0.277	-0.021	0.188	0.242
	*p*	0.379	0.024	0.617	0.212	0.997	0.053	0.384	0.100	0.787	0.138	0.871	0.112	0.862	0.108	0.906	0.278	0.161
FCVIQ	*r_s_*	-0.409^*^	-0.301	-0.499^**^	-0.372^*^	-0.397^*^	-0.394^*^	-0.459^**^	-0.197	-0.476^**^	-0.317	-0.471^**^	-0.321	-0.486^**^	-0.313	-0.477^**^	-0.296	-0.334^*^
	*p*	0.015	0.079	0.002	0.028	0.018	0.019	0.006	0.258	0.004	0.064	0.004	0.06	0.003	0.067	0.004	0.085	0.050

Cases were excluded pairwise. Significant results are shown in bold: *p ≤ 0.05, **p ≤ 0.01, ***p ≤ 0.001. Age and ICV were standardized to be included as covariates.

CC, corpus callosum; IFOF, inferior fronto-occipital fasciculus; ILF, inferior longitudinal fasciculus; OR, optic radiation; SLF, superior longitudinal fasciculus; d, dorsal; v, ventral; VOF, vertical occipital fasciculus; FrACT, Freiburg Visual Acuity Test; TVPS-4, Test of Visual Perceptual Skills, Fourth Edition; Beery, Beery-Buktenica Test of Visual-Motor Integration, Sixth Edition; FCVIQ, Flemish cerebral visual impairment questionnaire. *r_s_*, Spearman correlations interpreted as no or negligible correlation (<0.30), low (0.30–0.49), moderate (0.50–0.69), high (0.70–0.89), or very high (≥0.90) (Mukaka, 2012); *p*, *p*-value.

**Table 4.** Semi-partial Spearman Rank’s correlations corrected for age and ICV between the results of the visual assessments and the FC of the visual tracts.

	Fiber cross-section (FC)																	
Visual assessment		Occipital CC	Left IFOF	Right IFOF	Left ILF	Right ILF	Left OR	Right OR	Left SLF I	Right SLF I	Left SLF IId	Right SLF IId	Left SLF III	Right SLF III	Left SLF IIv	Right SLF IIv	Left VOF	Right VOF
FraCT	*r_s_*	-0.290	-0.142	-0.371*	-0.167	-0.205	-0.335*	-0.359*	-0.144	-0.377*	-0.143	-0.280	-0.192	-0.326	-0.157	-0.310	-0.075	-0.167
	*p*	0.086	0.407	0.026	0.329	0.231	0.046	0.031	0.402	0.024	0.406	0.098	0.261	0.052	0.361	0.066	0.664	0.330
Titmus Stereo Fly	*r_s_*	0.327	0.359*	0.393*	0.226	0.308	0.289	0.389*	0.204	0.086	0.138	0.228	0.196	0.248	0.166	0.208	0.249	0.420*
	*p*	0.052	0.032	0.018	0.186	0.067	0.087	0.019	0.232	0.618	0.424	0.182	0.253	0.145	0.335	0.223	0.143	0.011
TVPS-4 Visual Discrimination	*r_s_*	0.010	-0.032	0.183	0.010	0.112	0.046	0.146	-0.095	0.050	0.055	0.381*	0.045	0.362*	0.006	0.316	-0.157	0.009
	*p*	0.953	0.855	0.286	0.952	0.514	0.790	0.397	0.580	0.773	0.748	0.022	0.797	0.030	0.974	0.061	0.360	0.958
TVPS-4 Spatial Relationships	*r_s_*	0.150	0.132	0.171	0.178	0.135	0.154	0.158	0.161	0.015	0.165	0.275	0.142	0.267	0.138	0.217	0.041	0.121
	*p*	0.382	0.444	0.319	0.299	0.434	0.370	0.357	0.349	0.930	0.336	0.104	0.407	0.115	0.421	0.204	0.812	0.481
TVPS-4 Form Constancy	*r_s_*	0.209	0.043	0.227	0.073	0.142	0.145	0.246	0.075	0.126	0.066	0.326	0.091	0.324	0.052	0.276	-0.053	0.167
	*p*	0.222	0.802	0.182	0.673	0.409	0.398	0.147	0.663	0.464	0.700	0.052	0.599	0.054	0.763	0.104	0.758	0.331
TVPS-4 Visual Figure-Ground	*r_s_*	0.021	0.004	0.179	-0.068	0.104	0.015	0.154	0.005	-0.064	0.084	0.166	0.050	0.162	0.060	0.134	-0.190	0.082
	*p*	0.905	0.981	0.297	0.692	0.544	0.931	0.371	0.977	0.711	0.627	0.333	0.772	0.345	0.730	0.436	0.268	0.636
TVPS-4 Visual Closure	*r_s_*	0.107	0.028	0.220	0.053	0.133	0.103	0.247	0.191	0.018	0.082	0.105	0.085	0.158	0.077	0.102	-0.049	0.157
	*p*	0.533	0.872	0.196	0.759	0.441	0.552	0.147	0.264	0.918	0.636	0.544	0.623	0.357	0.657	0.555	0.777	0.360
Beery- Visuomotor integration	*r_s_*	0.159	0.417*	0.029	0.201	-0.065	0.396*	0.101	0.322	-0.136	0.245	-0.039	0.271	-0.015	0.265	-0.038	0.098	0.126
	*p*	0.361	0.013	0.869	0.247	0.712	0.018	0.563	0.059	0.435	0.156	0.825	0.116	0.931	0.124	0.828	0.574	0.471
FCVIQ	*r_s_*	-0.338*	-0.347*	-0.446**	-0.378*	-0.355*	-0.437**	-0.371*	-0.165	-0.357*	-0.372*	-0.424*	-0.362*	-0.483**	-0.342*	-0.443**	-0.265	-0.318
	*p*	0.047	0.041	0.007	0.025	0.036	0.009	0.028	0.343	0.035	0.028	0.011	0.033	0.003	0.045	0.008	0.125	0.063

Cases were excluded pairwise. Significant results are shown in bold: *p ≤ 0.05, **p ≤ 0.01, ***p ≤ 0.001. Age and ICV were standardized to be included as covariates.

CC, corpus callosum; IFOF, inferior fronto-occipital fasciculus; ILF, inferior longitudinal fasciculus; OR, optic radiation; SLF, superior longitudinal fasciculus; d, dorsal; v, ventral; VOF, vertical occipital fasciculus; FrACT, Freiburg Visual Acuity Test; TVPS-4, Test of Visual Perceptual Skills, Fourth Edition; Beery, Beery-Buktenica Test of Visual-Motor Integration, Sixth Edition; FCVIQ, Flemish cerebral visual impairment questionnaire. *r_s_*, Spearman correlations interpreted as no or negligible correlation (<0.30), low (0.30–0.49), moderate (0.50–0.69), high (0.70–0.89), or very high (≥0.90) (Mukaka, 2012); *p*, *p*-value.

**Table 5.** Spearman Rank’s correlations between the results of the visual assessments and the AFD of the visual tracts.

	Apparent fiber density (AFD)																	
Visual assessments		Occipital CC	Left IFOF	Right IFOF	Left ILF	Right ILF	Left OR	Right OR	Left SLF I	Right SLF I	Left SLF IId	Right SLF IId	Left SLF III	Right SLF III	Left SLF IIv	Right SLF IIv	Left VOF	Right VOF
FraCT	*r_s_*	-0.518^***^	-0.393^*^	-0.402^*^	-0.513^***^	-0.423^**^	-0.448^**^	-0.557^***^	-0.229	-0.412^*^	-0.386^*^	-0.406^*^	-0.304	-0.345^*^	-0.416^*^	-0.306	-0.412^*^	-0.534^***^
	*p*	0.001	0.018	0.015	0.001	0.010	0.006	<0.001	0.179	0.013	0.020	0.014	0.071	0.039	0.012	0.070	0.012	0.001
Titmus Stereo Fly	*r_s_*	0.324	0.452^**^	0.543^***^	0.385^*^	0.381^*^	0.336^*^	0.461^**^	0.251	0.350^*^	0.317	0.287	0.380^*^	0.193	0.432^**^	0.246	0.331^*^	0.571^***^
	*p*	0.054	0.006	0.001	0.020	0.022	0.045	0.005	0.140	0.036	0.060	0.090	0.022	0.260	0.009	0.148	0.048	<0.001
TVPS-4 Visual Discrimination	*r_s_*	0.253	0.329^*^	0.285	0.347^*^	0.443^**^	0.354^*^	0.336^*^	0.133	0.260	0.171	0.266	0.100	0.179	0.136	0.100	0.128	0.317
	*p*	0.137	0.050	0.092	0.038	0.007	0.034	0.045	0.438	0.126	0.320	0.117	0.562	0.297	0.427	0.560	0.456	0.060
TVPS-4 Spatial Relationships	*r_s_*	0.335^*^	0.370^*^	0.345^*^	0.377^*^	0.541^***^	0.406^**^	0.422^**^	0.139	0.136	0.195	0.209	0.263	0.163	0.243	0.116	0.258	0.497^**^
	*p*	0.046	0.026	0.039	0.023	0.001	0.014	0.010	0.419	0.428	0.254	0.221	0.121	0.341	0.153	0.500	0.129	0.002
TVPS-4 Form Constancy	*r_s_*	0.503^**^	0.329^*^	0.376^*^	0.391^*^	0.466^**^	0.408^*^	0.488^**^	0.169	0.332^*^	0.250	0.261	0.226	0.230	0.240	0.107	0.185	0.549^***^
	*p*	0.002	0.050	0.024	0.018	0.004	0.013	0.003	0.323	0.048	0.141	0.124	0.185	0.178	0.159	0.534	0.281	0.001
TVPS-4 Visual Figure-Ground	*r_s_*	0.369^*^	0.428^**^	0.395^*^	0.388^*^	0.540^***^	0.447^**^	0.508^**^	0.137	0.310	0.263	0.347^*^	0.247	0.293	0.288	0.283	0.193	0.360^*^
	*p*	0.027	0.009	0.017	0.019	0.001	0.006	0.002	0.425	0.066	0.121	0.038	0.146	0.083	0.088	0.095	0.261	0.031
TVPS-4 Visual Closure	*r_s_*	0.435^**^	0.249	0.305	0.358^*^	0.461^**^	0.460^**^	0.432^**^	0.236	0.212	0.196	0.314	0.248	0.333^*^	0.287	0.255	0.308	0.398^*^
	*p*	0.008	0.144	0.070	0.032	0.005	0.005	0.008	0.167	0.215	0.251	0.063	0.145	0.047	0.090	0.134	0.067	0.016
Beery- Visuomotor integration	*r_s_*	0.291	0.291	0.358^*^	0.421^*^	0.463^**^	0.403^*^	0.418^*^	0.178	-0.106	0.080	0.011	0.210	-0.070	0.245	-0.016	0.400^*^	0.575^***^
	*p*	0.090	0.090	0.035	0.012	0.005	0.016	0.012	0.305	0.545	0.647	0.949	0.225	0.690	0.157	0.928	0.017	<0.001
FCVIQ	*r_s_*	**-0.521** ^***^	-0.410*	**-0.558** ^***^	**-0.507** ^**^	**-0.486** ^**^	**-0.468** ^**^	**-0.541** ^***^	-0.245	**-0.357** ^*^	-0.166	**-0.499** ^**^	-0.278	**-0.376** ^*^	-0.317	**-0.382** ^*^	**-0.439** ^**^	**-0.502** ^**^
	*p*	0.001	0.014	<0.001	0.002	0.003	0.005	0.001	0.156	0.035	0.342	0.002	0.106	0.026	0.063	0.024	0.008	0.002

Cases were excluded pairwise. Significant results are shown in bold: *p ≤ 0.05, **p ≤ 0.01. ***p ≤ 0.001.CC, corpus callosum; IFOF, inferior fronto-occipital fasciculus; ILF, inferior longitudinal fasciculus; OR, optic radiation; SLF, superior longitudinal fasciculus; d, dorsal; v, ventral; VOF, vertical occipital fasciculus; FrACT, Freiburg Visual Acuity Test; TVPS-4, Test of Visual Perceptual Skills, Fourth Edition; Beery, Beery-Buktenica Test of Visual-Motor Integration, Sixth Edition; FCVIQ, Flemish cerebral visual impairment questionnaire. *r_s_*, Spearman correlations interpreted as no or negligible correlation (<0.30), low (0.30–0.49), moderate (0.50–0.69), high (0.70–0.89), or very high (≥0.90) (Mukaka, 2012); *p*, *p*-value.

## 4 Discussion

In this study, we conducted a comprehensive exploration of the micro- and macrostructural differences in WM properties of the visual system between children with left- and right-sided uCP, and their relation to specific visual outcomes, using fixel-based analysis. Our first objective was to compare the micro- and macrostructural properties of visual pathways between children with left- and right-sided uCP. We found that children with left-sided uCP have significantly lower AFD, FC, and FDC in the right SLF, IFOF, and OR, while children with right-sided uCP have lower fixel metrics in the left SLF only. Our second objective was to investigate the relation between visual outcomes and WM matter properties of the visual tracts across the entire uCP cohort. Our findings showed that different visual outcomes are correlated with several WM visual pathways, suggesting that visual functions are controlled by a complex neural network, rather than isolated tracts. Interestingly, our covariates analysis showed that only age was associated with the FC and FDC of the visual pathways, suggesting that in children with uCP, age-related changes have a stronger impact on macro- (i.e., bundle size) rather than microstructural WM properties of the visual pathway. While the primary analyses were exploratory and reported without correction for multiple comparisons, we also applied FDR correction for transparency, showing that several differences in FC and FDC between children with left- and right-sided uCP, and correlations between AFD and visual function, remained significant.

Since the motor system is functionally lateralized (i.e., the left hemisphere primarily controls the motor output of the right limbs), early brain damage in children with uCP causes motor impairments on the side of the body opposite to the most affected hemisphere (Martin, 2005). In the visual system, the relation between visual outcomes and brain damage is not so straightforward, since both the left and right primary visual cortex are required to integrate the input from the contralateral visual field (Braddick & Atkinson, 2011). Additionally, the presence of hemispheric specificity for visual functions is still not fully understood, making it more challenging to identify differences in visual pathways between children with left- and right-sided uCP (Crotti, Genoe, et al., 2024; Pagnozzi et al., 2020). In our previous research (Crotti, Ben Itzhak, et al., 2024), we found no difference in the WM lobar and total score of the semi-quantitative scale developed by Fiori et al. (2014) between children with left- and right-sided uCP, suggesting the need for more advanced techniques to explore whether differences in WM connectivity could be detected between groups. Therefore, in this exploratory study, we used fixel-based analysis, showing, for the first time, that children with left-sided uCP present with lower AFD, FC, and FDC in different WM tracts (SLF, IFOF, OR) of the right hemisphere, while children with right-sided uCP show lower fixel metrics limited to the left SLF. Our results on the OR are partially in line with our hypothesis based on previous findings (Araneda et al., 2022; Maiani et al., 2024), since we only found differences between groups in the right hemisphere for the FC and FDC, while previous research showed reduced voxel-metrics on the lesioned hemisphere (either right or left) compared to the non-lesioned hemisphere in children with uCP. Differences could be explained by the type of analysis performed (i.e., fixel-based rather than voxel-based metrics), highlighting the added value of performing CSD to enhance the accuracy of WM tractography. An additional explanation could be the presence of widespread (bilateral) lesions in our cohort and that the classification (i.e., right/left-sided uCP) is based on their clinical motor performance and not on the lesioned/non-lesioned hemisphere. Nevertheless, we found no significant differences in lobar or total WM scores (Fiori et al., 2014) between groups (Crotti, Ben Itzhak, et al., 2024), indicating that lesion extent was comparable across children with left- and right-sided uCP. Additionally, it is important to acknowledge that we did not have a control group; hence, comparisons with normative values of the fixel metrics of right and left hemispheres in school-age children without brain lesions were not possible to make. Nevertheless, the present study provides the first evidence of differences in WM properties of the right IFOF and bilateral SLF between children with right- and left-sided uCP, with the SLF I being the only WM tract showing differences between groups in both hemispheres at the micro- (AFD), macro (FC), and combined structural properties (FDC). Given the exploratory nature and novelty of our study, our explanations are presented as hypotheses that could guide future research. The SLF is a major WM tract connecting the frontal, parietal, and occipital lobes, divided into three branches: SLF I, SLF II (further divided into dorsal and ventral in the present study), and SLF III, which have different functions such as visuospatial attention and visuomotor coordination (SLF I), attention, working memory, and higher-order cognitive functions (SLF II), and language processing (SLF III) (Nakajima et al., 2020; Thiebaut de Schotten et al., 2011). Previous studies showed that a right-larger-than-left SLF volume was associated with faster visuospatial processing in right-handed participants without brain lesions, suggesting the relation between this tract and hemispheric specialization (Budisavljevic et al., 2017). Additional findings also showed that differences in SLF volume strongly correlated with hand preference (Howells et al., 2018). Given its relation with visuomotor, visuospatial processing, and hand lateralization (Budisavljevic et al., 2021; Thiebaut de Schotten et al., 2011), further studies on the micro- and macrostructural changes of the SLF in children with uCP could provide additional clinical insights in this neurodevelopmental disorder and its comorbidities. Remarkably, no significant differences in visual functions were observed between the groups as shown in our previous study (Crotti, Ortibus, Mailleux, et al., 2024), supporting the notion that hemispheric differences in WM properties of the visual pathways might not always result in differences in visual functions between children with left- and right-sided uCP. Furthermore, brain areas with damaged tissue might still be actively involved in visual processing, due to reorganization within the damaged cortex (Araneda et al., 2022; Grasso et al., 2020; Guzzetta, 2010).

The results of the correlation analysis partially align with our initial hypothesis that lower fixel metrics in distinct WM tracts would be associated with reduced specific visual outcomes in children with uCP. Our findings showed that, in children with uCP, lower results on specific visual assessments were related to lower values of the FDC, FC, and AFD of different visual tracts, which can indicate a reduction in the size of fiber bundles (FC), loss of fiber density (AFD), and/or a combination of both (FDC) (Raffelt et al., 2017). However, we also identified distinct moderate associations that could suggest that damage to specific visual tracts may be more related to distinct lower visual outcomes. Specifically, reduced *visual acuity* was associated with lower values of all three metrics of the right IFOF, OR, and SLF I, suggesting that, in children with uCP, reduced axonal density (AFD) and reduced bundle calibre (FC), especially in the right hemisphere, may impair the ability to perceive fine details. The OR connects the lateral geniculate nuclei of the thalami with the occipital cortex (Swienton & Thomas, 2014), and the relation between its damage and impaired visual acuity was already established in children with CP using DTI (Ceschin et al., 2015), and in preterm infants and children using CSD (Chandwani et al., 2022; Thompson et al., 2014). Notably, our study is consistent with the results of Araneda et al. (2022), reporting that although lesions in either hemisphere can impact the microstructural properties of the OR, the impact of the right hemispheric lesions is more pronounced due to its specialization in visuospatial functions. Our results further extend prior findings showing a relation between lower level of visual acuity and both reduced axonal density (microstructural) and reduced bundle calibre (macrostructural) of the IFOF and SLF I, and specifically microstructural properties of the occipital CC, bilateral ILF, and VOF. The ILF connects the occipital lobe to the anterior temporal lobe (Herbet et al., 2018), and the IFOF runs between the occipital and frontal lobes (Conner et al., 2018). Both WM tracts are part of the ventral visual stream and, according to previous findings, are involved in object recognition (Ortibus et al., 2012), reading, and visually-guided behaviors, functions for which a good level of visual acuity is required. Results of the VOF, which connects the dorsal and ventral visual pathways (Takemura et al., 2016), and of the occipital CC, which connects the bilateral occipital lobes via the splenium of the CC (Berlucchi, 2014), indicate that both reduced intra- and interhemispheric connectivity is related to lower visual acuity in children with uCP. Lower level of *stereoacuity* also correlated with several WM tracts, with the most significant results on the right VOF, strengthening the hypothesis that the right posterior parietal cortex and its connections to the temporal lobe via the VOF are critical for 3D perception, in line with previous findings in participants without brain lesions (Nishida et al., 2001; Oishi et al., 2018). For the *TVPS-4 subtests*, lower performance was related to lower values on all three metrics of the right SLF only, supporting the hypothesis of the right hemisphere specialization for visual-perceptual functions. Previous results, based on CSD, showed that in preterm infants damage to the SLF can impact visual attention, a relevant function in the performance of the TVPS-4 subtests which require the identification of a target figure among different options (Chandwani et al., 2021). Our results are also supported by the study of Galli et al. (2018) which found significant differences in the FA of the SLF between children with CP with and without visual-associative disorders, classified based on visual-perceptual and visuomotor integration tests. Notably, in our study, lower results on the TVPS-4 subtests also showed correlations with lower microstructural properties (AFD) of the WM visual pathways, including the occipital CC, right SLF, bilateral IFOF, ILF, OR, and VOF. Similarly, lower performance on the *VMI* was mostly correlated with lower AFD of the right IFOF, bilateral ILF, OR, and VOF, suggesting that, in children with uCP, reduced motor-free visual-perceptual and VMI functions show more associations with altered reduced axonal density rather than reduced calibre of the visual pathways. Results on relation to the ILF are consistent with previous findings, showing that children with impairment in visual perception and object recognition present with lower FA on the ILF (Ortibus et al., 2012). Nevertheless, we also showed that lower VMI performance is related to reduced calibre (FC) of the IFOF and OR, particularly of the left hemisphere. In a functional MRI study, specific activation in the left lingual gyrus and cuneus, which form the medial occipital lobe (Palejwala et al., 2021), was observed during a copy task performed by participants without brain lesions (Ferber et al., 2007). This finding suggests that the left visual network is involved in visual feedback and attentional shift processes, key functions needed to compare one’s copy to a target figure, as required in tasks like the VMI (Ferber et al., 2007). Lastly, impaired *functional vision* was the visual outcome reporting the most number of associations with both micro- (axonal density), macro- (reduced bundle calibre), and combined structural properties of the visual pathways, including the occipital CC, bilateral ILF, OR, and right IFOF and SLF. This result is not surprising, since the FCVIQ is a questionnaire assessing the use, in daily life, of different visual functions involving both the dorsal (SLF) and ventral stream (ILF, IFOF), with items related to visual attention and complex problem-solving, which require good inter- (occipital CC) and intrahemispheric connections (OR) (Parks & Madden, 2013). Remarkably, the right IFOF was the WM tract showing the strongest correlations with the FCVIQ, supporting earlier findings that damage to this tract (i.e., lower FA) was the most discriminant between participants with and without cerebral visual impairment (Bauer & Merabet, 2024). Since the IFOF is a long-range fiber bundle involved in several visual and cognitive functions (Englander et al., 2013), it can be particularly affected by early brain damage, resulting in higher impairments in functional vision in children with uCP. Overall, our analysis did not identify a single WM tract responsible for a specific visual outcome, highlighting that visual functions are controlled by a complex neural network. However, damage to the VOF, particularly in the right hemisphere, may warrant special attention to the assessment of stereoacuity, while damage to the IFOF, especially in the left hemisphere, of VMI. Additionally, understanding hemispheric specialization for visual functions is critical, since knowledge of the neurological correlates of specific visual functions can guide tailored interventions for children with left- or right-sided uCP. Taken together, our results suggest that reduced visual acuity, stereoacuity, and motor-free visual perceptual function are more associated with damage to right-hemisphere WM tracts, lower VMI abilities to the left visual pathways, while impairments in functional vision relate to both hemispheres.

### 4.1 Limitations

Some limitations of our study should also be noted. First, our sample did not include children with neurotypical development, preventing the possibility of comparing the properties of the WM visual pathways with a control group. As an additional consequence, imaging data from children with uCP, rather than age-matched children with neurotypical development, were used to generate the population template. Nevertheless, the inclusion of 10-flipped images and the exclusion of voxels with lesions ensured a symmetrical template with minimal influence of visible lesions. Secondly, in our correlation analysis, we did not apply corrections for multiple comparisons which can reduce the risk of false positive findings (Rothman, 1990). Given the novelty and exploratory nature of our study, we believe that it was appropriate to refrain from correcting for multiple comparisons in the primary analyses, as this could increase the risk of overlooking potentially meaningful associations. However, to ensure transparency, we additionally performed FDR correction and reported the results in the Supplementary Materials. Third, our study investigated the WM properties of the right and left hemispheres rather than investigating the most/least affected hemisphere in children with uCP. This approach was motivated by the inclusion of children with uCP with widespread bilateral lesions, where identifying the most affected WM visual tracts is not straightforward. Nevertheless, as discussed above, we controlled that the two groups did not differ in lesion extent. Lastly, we acknowledge that our sample size (*N* = 36) limited the possibility of applying more complex fixel-based analyses (i.e., segment-specific (Chandio et al., 2020)) and statistical models (i.e., regression with additional covariates) given the 17 tracts of interest (i.e., predictors), and 8 visual outcomes which would have potentially led to the risk of overfitting. To address this constraint, we opted to use bundle-averaged FBA fixel-metrics and more simple statistical methods (i.e., correlations) to perform an exploratory study.

### 4.2 Strengths and future directions

Despite the limitations and the exploratory nature of our research, our results are promising, since they show the value of applying CSD bundle averaged FBA analysis to independently investigate the micro- and macrostructural WM properties of the visual system to achieve a deeper understanding of the neural correlates of visual functions in children with uCP. Particularly, based on our correlation analysis, future studies with a larger number of participants could refine regression models by focusing on the strongest relation identified between specific WM tracts and visual outcomes. To address concerns related to multiple comparisons and statistical power, future work might also consider employing multivariate approaches. Longitudinal studies would be valuable to examine how these relationships evolve with age. This work could better identify potential biomarkers based on WM integrity to predict visual impairments in children with uCP, allowing for early and tailored support. Additionally, future research could explore the added value of performing a segment-specific analysis for each visual tract (i.e., BUAN framework (Chandio et al., 2020)) to achieve further spatial precision.

## 5 Conclusion

In conclusion, using advanced dMRI fixel-based analysis, we first showed hemisphere-specific differences between children with left- and right-sided uCP in the visual pathways, particularly in the SLF. Secondly, we found distinct associations between different visual outcomes and micro- (AFD), macro- (FC), and combined (FDC) structural properties of the WM visual pathways in children with uCP. Our results provide new insights into the structure-function relation between the micro- and macrostructural properties of the WM tracts and visual outcomes in children with uCP, which could guide future research. Specifically, longitudinal studies, starting from infancy, could assess how these relations emerge, potentially revealing developmental windows for intervention that maximize visual and motor improvements in children with uCP.

## Supplementary Material

Supplementary Material

## Data Availability

Data can be made available from the corresponding author upon reasonable request.

## References

[IMAG.a.122-b1] Andersen, S. M., Rapcsak, S. Z., & Beeson, P. M. (2010). Cost function masking during normalization of brains with focal lesions: Still a necessity? NeuroImage, 53(1), 78–84. 10.1016/j.neuroimage.2010.06.003 20542122 PMC2938189

[IMAG.a.122-b2] Andersson, J. L. R., Skare, S., & Ashburner, J. (2003). How to correct susceptibility distortions in spin-echo echo-planar images: Application to diffusion tensor imaging. NeuroImage, 20(2), 870–888. 10.1016/S1053-8119(03)00336-7 14568458

[IMAG.a.122-b3] Andersson, J. L. R., & Sotiropoulos, S. N. (2016). An integrated approach to correction for off-resonance effects and subject movement in diffusion MR imaging. NeuroImage, 125, 1063–1078. 10.1016/j.neuroimage.2015.10.019 26481672 PMC4692656

[IMAG.a.122-b4] Araneda, R., Ebner-Karestinos, D., Dricot, L., Herman, E., Hatem, S. M., Friel, K. M., Gordon, A. M., & Bleyenheuft, Y. (2022). Impact of early brain lesions on the optic radiations in children with cerebral palsy. Frontiers in Neuroscience, 16, 924938. 10.3389/fnins.2022.924938 36278011 PMC9583910

[IMAG.a.122-b5] Ard, T., Bienkowski, M. S., Liew, S.-L., Sepehrband, F., Yan, L., & Toga, A. W. (2022). Integrating data directly into publications with augmented reality and web-based technologies—Schol-AR. Scientific Data, 9(1), 298. 10.1038/s41597-022-01426-y

[IMAG.a.122-b6] Avants, B. B., Tustison, N. J., Song, G., Cook, P. A., Klein, A., & Gee, J. C. (2011). A reproducible evaluation of ANTs similarity metric performance in brain image registration. NeuroImage, 54(3), 2033–2044. 10.1016/j.neuroimage.2010.09.025 20851191 PMC3065962

[IMAG.a.122-b7] Bach, M. (1996). The Freiburg Visual Acuity Test—Automatic measurement of visual acuity. Optometry and Vision Science, 73(1), 49–53. https://journals.lww.com/optvissci/Fulltext/1996/01000/The_Freiburg_Visual_Acuity_Test_Automatic.8.aspx 8867682 10.1097/00006324-199601000-00008

[IMAG.a.122-b8] Bauer, C. M., & Merabet, L. B. (2024). Aberrant white matter development in cerebral visual impairment: A proposed mechanism for visual dysfunction following early brain injury. Journal of Integrative Neuroscience, 23(1), 1. 10.31083/j.jin2301001 38287851 PMC12434583

[IMAG.a.122-b9] Bedetti, C., arnaudbore, Guay, S., Carlin, J., Nick, Dastous, A., Joseph, M., jstaph, Routier, A., Kastman, E., Stojic, H., Isla, & Callenberg, K. (2022). UNFmontreal/Dcm2Bids: 2.1.7 (Version 2.1.7) [Computer software]. Zenodo. 10.5281/zenodo.6596007

[IMAG.a.122-b10] Beery, K. E., Buktenica, N. A., & Beery, N. A. (2010). Developmental test of Visual-Motor Integration, Sixth edition, Revised (VMI). https://scholar.google.com/scholar?hl=en&q=Beery+KE%2C+Buktenica+NA%2C+Beery+NA.+Developmental+Test+of+Visual%E2%80%90Motor+Integration%2C+Sixth+Edition%2C+Revised+%28VMI%29.+Developmental+Test+of+Visual%E2%80%90Motor+Integration%2C+Sixth+Edition%2C+Revised+%28VMI%29+2010

[IMAG.a.122-b11] Ben Itzhak, N., Vancleef, K., Franki, I., Laenen, A., Wagemans, J., & Ortibus, E. (2021). Quantifying visuoperceptual profiles of children with cerebral visual impairment. Child Neuropsychology, 27(8), 995–1023. 10.1080/09297049.2021.1915265 33944679

[IMAG.a.122-b12] Bender, R., & Lange, S. (2001). Adjusting for multiple testing—When and how? Journal of Clinical Epidemiology, 54(4), 343–349. 10.1016/s0895-4356(00)00314-0 11297884

[IMAG.a.122-b13] Benjamini, Y., & Hochberg, Y. (1995). Controlling the false discovery rate: A practical and powerful approach to multiple testing. Journal of the Royal Statistical Society: Series B (Methodological), 57(1), 289–300. 10.1111/j.2517-6161.1995.tb02031.x

[IMAG.a.122-b14] Berelowitz, S., & Franzsen, D. (2021). Visual perceptual deficits in different types of cerebral palsy. South African Journal of Occupational Therapy, 51(1). 10.17159/2310-3833/2021/vol51n1a4

[IMAG.a.122-b15] Berlucchi, G. (2014). Visual interhemispheric communication and callosal connections of the occipital lobes. Cortex: A Journal Devoted to the Study of the Nervous System and Behavior, 56, 1–13. 10.1016/j.cortex.2013.02.001 23489777

[IMAG.a.122-b16] Braddick, O., & Atkinson, J. (2011). Development of human visual function. Vision Research, 51(13), 1588–1609. 10.1016/j.visres.2011.02.018 21356229

[IMAG.a.122-b17] Brown, T., & Peres, L. (2018). An overview and critique of the Test of Visual Perception Skills—Fourth edition (TVPS-4). Hong Kong Journal of Occupational Therapy, 31(2), 59–68. 10.1177/1569186118793847 30643493 PMC6322110

[IMAG.a.122-b18] Budisavljevic, S., Castiello, U., & Begliomini, C. (2021). Handedness and white matter networks. The Neuroscientist: A Review Journal Bringing Neurobiology, Neurology and Psychiatry, 27(1), 88–103. 10.1177/1073858420937657 32723129

[IMAG.a.122-b19] Budisavljevic, S., Dell’Acqua, F., Zanatto, D., Begliomini, C., Miotto, D., Motta, R., & Castiello, U. (2017). Asymmetry and structure of the fronto-parietal networks underlie visuomotor processing in humans. Cerebral Cortex (New York, N.Y.: 1991), 27(2), 1532–1544. 10.1093/cercor/bhv348 26759477

[IMAG.a.122-b20] Burtner, P. A., Dukeminier, A., Ben, L., Qualls, C., & Scott, K. (2006). Visual perceptual skills and related school functions in children with hemiplegic cerebal palsy. New Zealand Journal of Occupational Therapy, 53(1), 24+. 10.3233/nre-2009-0459

[IMAG.a.122-b21] Ceschin, R., Lee, V. K., Schmithorst, V., & Panigrahy, A. (2015). Regional vulnerability of longitudinal cortical association connectivity. NeuroImage: Clinical, 9, 322–337. 10.1016/j.nicl.2015.08.021 26509119 PMC4588423

[IMAG.a.122-b22] Chandio, B. Q., Risacher, S. L., Pestilli, F., Bullock, D., Yeh, F.-C., Koudoro, S., Rokem, A., Harezlak, J., & Garyfallidis, E. (2020). Bundle analytics, a computational framework for investigating the shapes and profiles of brain pathways across populations. Scientific Reports, 10(1), 17149. 10.1038/s41598-020-74054-4 33051471 PMC7555507

[IMAG.a.122-b23] Chandwani, R., Harpster, K., Kline, J. E., Mehta, V., Wang, H., Merhar, S. L., Schwartz, T. L., & Parikh, N. A. (2022). Brain microstructural antecedents of visual difficulties in infants born very preterm. NeuroImage: Clinical, 34, 102987. 10.1016/j.nicl.2022.102987 35290855 PMC8918861

[IMAG.a.122-b24] Chandwani, R., Kline, J. E., Harpster, K., Tkach, J., Parikh, N. A., & Group, T. C. I. N. E. P. S. (CINEPS). (2021). Early micro- and macrostructure of sensorimotor tracts and development of cerebral palsy in high risk infants. Human Brain Mapping, 42(14), 4708–4721. 10.1002/hbm.25579 34322949 PMC8410533

[IMAG.a.122-b25] Cloutman, L. L. (2013). Interaction between dorsal and ventral processing streams: Where, when and how? Brain and Language, 127(2), 251–263. 10.1016/j.bandl.2012.08.003 22968092

[IMAG.a.122-b26] Colenbrander, A. (2005). Visual functions and functional vision. International Congress Series, 1282, 482–486. 10.1016/j.ics.2005.05.002

[IMAG.a.122-b27] Conner, A. K., Briggs, R. G., Sali, G., Rahimi, M., Baker, C. M., Burks, J. D., Glenn, C. A., Battiste, J. D., & Sughrue, M. E. (2018). A connectomic atlas of the human cerebrum—Chapter 13: Tractographic description of the inferior fronto-occipital fasciculus. Operative Neurosurgery, 15(Suppl. 1), S436–S443. 10.1093/ons/opy267 30260438 PMC6890527

[IMAG.a.122-b28] Crotti, M., Ben Itzhak, N., Mailleux, L., Kleeren, L., Decraene, L., Leenaerts, N., Lubián-Gutiérrez, M., Feys, H., & Ortibus, E. (2024). Seeing the unseen: The neurodevelopmental factors related to visual impairments in children with unilateral cerebral palsy. SSRN Scholarly Paper No. 4935727. 10.2139/ssrn.4935727 41734651

[IMAG.a.122-b29] Crotti, M., Genoe, S., Ben Itzhak, N., Mailleux, L., & Ortibus, E. (2024). The relation between neuroimaging and visual impairment in children and adolescents with cerebral palsy: A systematic review. Brain and Development, 46(2), 75–92. 10.1016/j.braindev.2023.11.002 38016876

[IMAG.a.122-b30] Crotti, M., Ortibus, E., Ben Itzhak, N., Kleeren, L., Decraene, L., Leenaerts, N., Feys, H., & Mailleux, L. (2024). The relation between visual functions, functional vision, and bimanual function in children with unilateral cerebral palsy. Research in Developmental Disabilities, 152, 104792. 10.1016/j.ridd.2024.104792 39018791

[IMAG.a.122-b31] Crotti, M., Ortibus, E., Mailleux, L., Decraene, L., Kleeren, L., & Itzhak, N. B. (2024). Visual, perceptual functions, and functional vision in children with unilateral cerebral palsy compared to children with neurotypical development. Developmental Medicine & Child Neurology, 66(8), 1084–1095. 10.1111/dmcn.15842 38269438

[IMAG.a.122-b32] Cruz, A. D. L., Morale, S. E., Jost, R. M., Kelly, K. R., & Birch, E. E. (2016). Modified test protocol improves sensitivity of the Stereo Fly Test. The American Orthoptic Journal, 66(1), 122. 10.3368/aoj.66.1.122 27799586 PMC6051409

[IMAG.a.122-b33] Decraene, L., Orban de Xivry, J.-J., Kleeren, L., Crotti, M., Verheyden, G., Ortibus, E., Feys, H., Mailleux, L., & Klingels, K. (2023). In-depth quantification of bimanual coordination using the Kinarm exoskeleton robot in children with unilateral cerebral palsy. Journal of Neuroengineering and Rehabilitation, 20(1), 154. 10.1186/s12984-023-01278-6 37951867 PMC10640737

[IMAG.a.122-b34] Dhollander, T., Clemente, A., Singh, M., Boonstra, F., Civier, O., Duque, J. D., Egorova, N., Enticott, P., Fuelscher, I., Gajamange, S., Genc, S., Gottlieb, E., Hyde, C., Imms, P., Kelly, C., Kirkovski, M., Kolbe, S., Liang, X., Malhotra, A.,… Caeyenberghs, K. (2021). Fixel-based analysis of diffusion MRI: Methods, applications, challenges and opportunities. NeuroImage, 241, 118417. 10.1016/j.neuroimage.2021.118417 34298083

[IMAG.a.122-b35] Dimond, D., Rohr, C. S., Smith, R. E., Dhollander, T., Cho, I., Lebel, C., Dewey, D., Connelly, A., & Bray, S. (2020). Early childhood development of white matter fiber density and morphology. NeuroImage, 210, 116552. 10.1016/j.neuroimage.2020.116552 31972280

[IMAG.a.122-b36] Dufresne, D., Dagenais, L., & Shevell, M. I. (2014). Spectrum of visual disorders in a population-based cerebral palsy cohort. Pediatric Neurology, 50(4), 324–328. 10.1016/j.pediatrneurol.2013.11.022 24468636

[IMAG.a.122-b37] Duke, R. E., Nwachukuw, J., Torty, C., Okorie, U., Kim, M. J., Burton, K., Gilbert, C., & Bowman, R. (2022). Visual impairment and perceptual visual disorders in children with cerebral palsy in Nigeria. The British Journal of Ophthalmology, 106(3), 427–434. 10.1136/bjophthalmol-2020-317768 33268343

[IMAG.a.122-b38] Dutton, G. N., McKillop, E. C. A., & Saidkasimova, S. (2006). Visual problems as a result of brain damage in children. The British Journal of Ophthalmology, 90(8), 932–933. 10.1136/bjo.2006.095349 16854832 PMC1857187

[IMAG.a.122-b39] Ego, A., Lidzba, K., Brovedani, P., Belmonti, V., Gonzalez-Monge, S., Boudia, B., Ritz, A., & Cans, C. (2015). Visual-perceptual impairment in children with cerebral palsy: A systematic review. Developmental Medicine & Child Neurology, 57, 46–51. 10.1111/dmcn.12687 25690117

[IMAG.a.122-b40] Eliasson, A.-C., Krumlinde-Sundholm, L., Rösblad, B., Beckung, E., Arner, M., Ohrvall, A.-M., & Rosenbaum, P. (2006). The Manual Ability Classification System (MACS) for children with cerebral palsy: Scale development and evidence of validity and reliability. Developmental Medicine and Child Neurology, 48(7), 549–554. 10.1017/S0012162206001162 16780622

[IMAG.a.122-b41] Englander, Z. A., Pizoli, C. E., Batrachenko, A., Sun, J., Worley, G., Mikati, M. A., Kurtzberg, J., & Song, A. W. (2013). Diffuse reduction of white matter connectivity in cerebral palsy with specific vulnerability of long range fiber tracts. NeuroImage: Clinical, 2, 440–447. 10.1016/j.nicl.2013.03.006 24179798 PMC3777769

[IMAG.a.122-b42] Fabbro, L., Scuoteguazza-Filho, M., Moribe, E. K., Rodrigues, L., Oliveira da Silva, I. A., Konichi-Dias, R. L., Silva Muniz, D. N., Fabbro, N., Pacharone Bertolini Bidinotto, D. N., & Bidinotto, L. T. (2020). Impact of visual perception restoration in quality of life in early childhood. International Journal of Research Studies in Medical and Health Sciences, 5(5), 15–19. 10.22259/ijrsmhs.0505004

[IMAG.a.122-b43] Farassat, N., Jehle, V., Heinrich, S. P., Lagrèze, W. A., & Bach, M. (2024). The Freiburg Acuity Test in preschool children: Testability, test-retest variability, and comparison with LEA symbols. Translational Vision Science & Technology, 13(3), 14. 10.1167/tvst.13.3.14 PMC10959192 38502142

[IMAG.a.122-b44] Farquharson, S., Tournier, J.-D., Calamante, F., Fabinyi, G., Schneider-Kolsky, M., Jackson, G. D., & Connelly, A. (2013). White matter fiber tractography: Why we need to move beyond DTI. Journal of Neurosurgery, 118(6), 1367–1377. 10.3171/2013.2.JNS121294 23540269

[IMAG.a.122-b45] Fazzi, E., Signorini, S. G., Piana, R., Bertone, C., Misefari, W., Galli, J., Balottin, U., & Bianchi, P. E. (2012). Neuro-ophthalmological disorders in cerebral palsy: Ophthalmological, oculomotor, and visual aspects. Developmental Medicine & Child Neurology, 54(8), 730–736. 10.1111/j.1469-8749.2012.04324.x 22712803

[IMAG.a.122-b46] Ferber, S., Mraz, R., Baker, N., & Graham, S. J. (2007). Shared and differential neural substrates of copying versus drawing: A functional magnetic resonance imaging study. NeuroReport, 18(11), 1089. 10.1097/WNR.0b013e3281ac2143 17589305

[IMAG.a.122-b47] Fiori, S., Cioni, G., Klingels, K., Ortibus, E., Van Gestel, L., Rose, S., Boyd, R. N., Feys, H., & Guzzetta, A. (2014). Reliability of a novel, semi-quantitative scale for classification of structural brain magnetic resonance imaging in children with cerebral palsy. Developmental Medicine & Child Neurology, 56(9), 839–845. 10.1111/dmcn.12457 24750109

[IMAG.a.122-b48] Fischl, B. (2012). FreeSurfer. NeuroImage, 62(2), 774–781. 10.1016/j.neuroimage.2012.01.021 22248573 PMC3685476

[IMAG.a.122-b49] *FreeSurferWiki, 2020*. (n.d.). https://surfer.nmr.mgh.harvard.edu/fswiki/recon-all

[IMAG.a.122-b50] Galli, J., Ambrosi, C., Micheletti, S., Merabet, L. B., Pinardi, C., Gasparotti, R., & Fazzi, E. (2018). White matter changes associated with cognitive visual dysfunctions in children with cerebral palsy: A diffusion tensor imaging study. Journal of Neuroscience Research, 96(11), 1766–1774. 10.1002/jnr.24307 30027677

[IMAG.a.122-b51] Gorrie, F., Goodall, K., Rush, R., & Ravenscroft, J. (2019). Towards population screening for Cerebral Visual Impairment: Validity of the five questions and the CVI questionnaire. PLoS One, 14(3), e0214290. 10.1371/journal.pone.0214290 30913240 PMC6435113

[IMAG.a.122-b52] Graham, H. K., Rosenbaum, P., Paneth, N., Dan, B., Lin, J.-P., Damiano, D. L., Becher, J. G., Gaebler-Spira, D., Colver, A., Reddihough, D. S., Crompton, K. E., & Lieber, R. L. (2016). Cerebral palsy. Nature Reviews: Disease Primers, 2, 15082. 10.1038/nrdp.2015.82 PMC9619297 27188686

[IMAG.a.122-b53] Grasso, P. A., Gallina, J., & Bertini, C. (2020). Shaping the visual system: Cortical and subcortical plasticity in the intact and the lesioned brain. Neuropsychologia, 142, 107464. 10.1016/j.neuropsychologia.2020.107464 32289349

[IMAG.a.122-b54] Guzzetta, A. (2010). Plasticity of the visual system after congenital brain damage: A few weeks can matter. Developmental Medicine & Child Neurology, 52(8), 699–699. 10.1111/j.1469-8749.2010.03678.x 20477834

[IMAG.a.122-b55] Guzzetta, A., D’acunto, G., Rose, S., Tinelli, F., Boyd, R., & Cioni, G. (2010). Plasticity of the visual system after early brain damage. Developmental Medicine & Child Neurology, 52(10), 891–900. 10.1111/j.1469-8749.2010.03710.x 20561008

[IMAG.a.122-b56] Hellige, J. B., Laeng, B., & Michimata, C. (2010). Processing asymmetries in the visual system. In K. Hugdahl & R. Westerhausen (Eds.), The two halves of the brain: Information processing in the cerebral hemispheres (pp. 379–415). MIT Press. 10.7551/mitpress/9780262014137.003.0279

[IMAG.a.122-b57] Herbet, G., Zemmoura, I., & Duffau, H. (2018). Functional anatomy of the inferior longitudinal fasciculus: From historical reports to current hypotheses. Frontiers in Neuroanatomy, 12, 77. 10.3389/fnana.2018.00077 30283306 PMC6156142

[IMAG.a.122-b58] Himmelmann, K., Horber, V., De La Cruz, J., Horridge, K., Mejaski-Bosnjak, V., Hollody, K., Krägeloh-Mann, I., & SCPE Working Group. (2017). MRI classification system (MRICS) for children with cerebral palsy: Development, reliability, and recommendations. Developmental Medicine and Child Neurology, 59(1), 57–64. 10.1111/dmcn.13166 27325153

[IMAG.a.122-b59] Hinkle, D. E., Wiersma, W., & Jurs, S. G. (2003). Applied statistics for the behavioral sciences (Vol. 663). Houghton Mifflin college division. 10.3102/10769986015001084

[IMAG.a.122-b60] Holladay, J. T. (2004). Visual acuity measurements. Journal of Cataract & Refractive Surgery, 30(2), 287. 10.1016/j.jcrs.2004.01.014 15030802

[IMAG.a.122-b61] Howells, H., Thiebaut de Schotten, M., Dell’Acqua, F., Beyh, A., Zappalà, G., Leslie, A., Simmons, A., Murphy, D. G., & Catani, M. (2018). Frontoparietal tracts linked to lateralized hand preference and manual specialization. Cerebral Cortex (New York, NY), 28(7), 1–13. 10.1093/cercor/bhy040 PMC6005057 29688293

[IMAG.a.122-b62] Hyde, C., Fuelscher, I., Enticott, P. G., Jones, D. K., Farquharson, S., Silk, T. J., Williams, J., & Caeyenberghs, K. (2018). White matter organization in developmental coordination disorder: A pilot study exploring the added value of constrained spherical deconvolution. NeuroImage: Clinical, 21, 101625. 10.1016/j.nicl.2018.101625 30552074 PMC6411781

[IMAG.a.122-b63] Jenkinson, M., Beckmann, C. F., Behrens, T. E. J., Woolrich, M. W., & Smith, S. M. (2012). FSL. NeuroImage, 62(2), 782–790. 10.1016/j.neuroimage.2011.09.015 21979382

[IMAG.a.122-b64] Jeurissen, B., Leemans, A., Tournier, J.-D., Jones, D. K., & Sijbers, J. (2013). Investigating the prevalence of complex fiber configurations in white matter tissue with diffusion magnetic resonance imaging. Human Brain Mapping, 34(11), 2747–2766. 10.1002/hbm.22099 22611035 PMC6870534

[IMAG.a.122-b65] Jeurissen, B., Tournier, J.-D., Dhollander, T., Connelly, A., & Sijbers, J. (2014). Multi-tissue constrained spherical deconvolution for improved analysis of multi-shell diffusion MRI data. NeuroImage, 103, 411–426. 10.1016/j.neuroimage.2014.07.061 25109526

[IMAG.a.122-b66] Jitsuishi, T., Hirono, S., Yamamoto, T., Kitajo, K., Iwadate, Y., & Yamaguchi, A. (2020). White matter dissection and structural connectivity of the human vertical occipital fasciculus to link vision-associated brain cortex. Scientific Reports, 10(1), 820. 10.1038/s41598-020-57837-7 31965011 PMC6972933

[IMAG.a.122-b67] Kellner, E., Dhital, B., Kiselev, V. G., & Reisert, M. (2016). Gibbs-ringing artifact removal based on local subvoxel-shifts. Magnetic Resonance in Medicine, 76(5), 1574–1581. 10.1002/mrm.26054 26745823

[IMAG.a.122-b68] Leppink, J. (2018). Analysis of Covariance (ANCOVA) vs. Moderated Regression (MODREG): Why the interaction matters. Health Professions Education, 4(3), 225–232. 10.1016/j.hpe.2018.04.001

[IMAG.a.122-b69] Maiani, M., Hilderley, A., Lebel, C., Geeraert, B., Carlson, H., & Kirton, A. (2024). Bilateral differences in structural connectivity of the afferent visual pathways of children with perinatal stroke. Aperture Neuro, 4, 1–13. 10.52294/001c.123922

[IMAG.a.122-b70] Mailleux, L., Decraene, L., Kalkantzi, A., Kleeren, L., Crotti, M., Campenhout, A. V., Verheyden, G., Ortibus, E., Green, D., Klingels, K., & Feys, H. (2024). Spatiotemporal coordination in children with unilateral cerebral palsy: Insights from a bimanual goal-directed task. European Journal of Paediatric Neurology: EJPN: Official Journal of the European Paediatric Neurology Society, 53, 73–87. 10.1016/j.ejpn.2024.10.003 39418827

[IMAG.a.122-b72] Martin, J. H. (2005). The corticospinal system: From development to motor control. The Neuroscientist, 11(2), 161–173. 10.1177/1073858404270843 15746384

[IMAG.a.122-b71] Martin, N. A. (2017). Test of Visual Perceptual Skills (4th ed). Novato, CA: Academic Therapy Publications. https://scholar.google.com/scholar_lookup?title=Test%20of%20Visual%20Perceptual%20Skills&author=N.%20A.%20Martin&publication_year=2017&

[IMAG.a.122-b73] Martín-Signes, M., Chica, A. B., Bartolomeo, P., & Thiebaut de Schotten, M. (2024). Streams of conscious visual experience. Communications Biology, 7(1), 1–7. 10.1038/s42003-024-06593-9 39068236 PMC11283449

[IMAG.a.122-b74] McCane, S. J. (2006). Test review: Motor-free visual perception test. Journal of Psychoeducational Assessment, 24(3), 265–272. 10.1177/0734282906286339

[IMAG.a.122-b75] Moganeswari, D., Thomas, J., Srinivasan, K., & Jacob, G. P. (2015). Test re-test reliability and validity of different visual acuity and stereoacuity charts used in preschool children. Journal of Clinical and Diagnostic Research: JCDR, 9(11), NC01. 10.7860/JCDR/2015/14407.6747 PMC4668442 26675120

[IMAG.a.122-b76] Mori, S., Crain, B. J., Chacko, V. P., & Van Zijl, P. C. M. (1999). Three-dimensional tracking of axonal projections in the brain by magnetic resonance imaging. Annals of Neurology, 45(2), 265–269. 10.1002/1531-8249(199902)45:2<265::AID-ANA21>3.0.CO;2-3 9989633

[IMAG.a.122-b77] Mukaka, M. M. (2012). Statistics corner: A guide to appropriate use of correlation coefficient in medical research. Malawi Medical Journal: The Journal of Medical Association of Malawi, 24(3), 69–71. 10.4314/mmj.v20i1.10949 23638278 PMC3576830

[IMAG.a.122-b78] Nakajima, R., Kinoshita, M., Shinohara, H., & Nakada, M. (2020). The superior longitudinal fascicle: Reconsidering the fronto-parietal neural network based on anatomy and function. Brain Imaging and Behavior, 14(6), 2817–2830. 10.1007/s11682-019-00187-4 31468374

[IMAG.a.122-b79] Nishida, Y., Hayashi, O., Iwami, T., Kimura, M., Kani, K., Ito, R., Shiino, A., & Suzuki, M. (2001). Stereopsis-processing regions in the human parieto-occipital cortex. NeuroReport, 12(10), 2259–2263. https://journals.lww.com/neuroreport/Fulltext/2001/07200/Stereopsis_processing_regions_in_the_human.43.aspx 11447346 10.1097/00001756-200107200-00043

[IMAG.a.122-b80] Oishi, H., Takemura, H., Aoki, S. C., Fujita, I., & Amano, K. (2018). Microstructural properties of the vertical occipital fasciculus explain the variability in human stereoacuity. Proceedings of the National Academy of Sciences of the United States of America, 115(48), 12289–12294. 10.1073/pnas.1804741115 30429321 PMC6275509

[IMAG.a.122-b81] Ortibus, E., Laenen, A., Verhoeven, J., De Cock, P., Casteels, I., Schoolmeesters, B., Buyck, A., & Lagae, L. (2011). Screening for cerebral visual impairment: Value of a CVI questionnaire. Neuropediatrics, 42(4), 138–147. 10.1055/s-0031-1285908 21913154

[IMAG.a.122-b82] Ortibus, E., Verhoeven, J., Sunaert, S., Casteels, I., Cock, P., & Lagae, L. (2012). Integrity of the inferior longitudinal fasciculus and impaired object recognition in children: A diffusion tensor imaging study: ILF and Object Recognition in Children. Developmental Medicine & Child Neurology, 54(1), 38–43. 10.1111/j.1469-8749.2011.04147.x 22171928

[IMAG.a.122-b83] Pagnozzi, A. M., Pannek, K., Fripp, J., Fiori, S., Boyd, R. N., & Rose, S. (2020). Understanding the impact of bilateral brain injury in children with unilateral cerebral palsy. Human Brain Mapping, 41(10), 2794–2807. 10.1002/hbm.24978 32134174 PMC7294067

[IMAG.a.122-b84] Palejwala, A. H., Dadario, N. B., Young, I. M., O’Connor, K., Briggs, R. G., Conner, A. K., O’Donoghue, D. L., & Sughrue, M. E. (2021). Anatomy and white matter connections of the lingual gyrus and cuneus. World Neurosurgery, 151, e426–e437. 10.1016/j.wneu.2021.04.050 33894399

[IMAG.a.122-b85] Pannek, K., Fripp, J., George, J. M., Fiori, S., Colditz, P. B., Boyd, R. N., & Rose, S. E. (2018). Fixel-based analysis reveals alterations is brain microstructure and macrostructure of preterm-born infants at term equivalent age. NeuroImage: Clinical, 18, 51–59. 10.1016/j.nicl.2018.01.003 29868441 PMC5984576

[IMAG.a.122-b86] Parks, E. L., & Madden, D. J. (2013). Brain connectivity and visual attention. Brain Connectivity, 3(4), 317–338. 10.1089/brain.2012.0139 23597177 PMC3749701

[IMAG.a.122-b87] Peters, B. D., Szeszko, P. R., Radua, J., Ikuta, T., Gruner, P., DeRosse, P., Zhang, J.-P., Giorgio, A., Qiu, D., Tapert, S. F., Brauer, J., Asato, M. R., Khong, P. L., James, A. C., Gallego, J. A., & Malhotra, A. K. (2012). White matter development in adolescence: Diffusion tensor imaging and meta-analytic results. Schizophrenia Bulletin, 38(6), 1308–1317. 10.1093/schbul/sbs054 22499780 PMC3494037

[IMAG.a.122-b88] Petri, S., & Tinelli, F. (2023). Visual impairment and periventricular leukomalacia in children: A systematic review. Research in Developmental Disabilities, 135, 104439. 10.1016/j.ridd.2023.104439 36796269

[IMAG.a.122-b89] Pueyo, V., García-Ormaechea, I., González, I., Ferrer, C., de la Mata, G., Duplá, M., Orós, P., & Andres, E. (2014). Development of the Preverbal Visual Assessment (PreViAs) questionnaire. Early Human Development, 90(4), 165–168. 10.1016/j.earlhumdev.2014.01.012 24508330

[IMAG.a.122-b90] R Core Team. (2021). R: A language and environment for statistical computing. https://www.r-project.org/

[IMAG.a.122-b91] Radwan, A., & Sunaert, S. (2024). Treanus/KUL_NIS [Shell]. https://github.com/treanus/KUL_NIS (Original work published 2018)

[IMAG.a.122-b92] Radwan, A., Sunaert, S., Schilling, K., Descoteaux, M., Landman, B. A., Vandenbulcke, M., Theys, T., Dupont, P., & Emsell, L. (2021). An atlas of white matter anatomy, its variability, and reproducibility based on Constrained Spherical Deconvolution of diffusion MRI. Neuroscience, 254, 119029. 10.1016/j.neuroimage.2022.119029 PMC10265547 35231632

[IMAG.a.122-b93] Raffelt, D. A., Tournier, J.-D., Smith, R. E., Vaughan, D. N., Jackson, G., Ridgway, G. R., & Connelly, A. (2017). Investigating white matter fibre density and morphology using fixel-based analysis. NeuroImage, 144(Pt A), 58–73. 10.1016/j.neuroimage.2016.09.029 27639350 PMC5182031

[IMAG.a.122-b94] Rai, Y., Chaturvedi, S., Paliwal, V. K., Goyal, P., Chourasia, A., Singh Rathore, R. K., Yadav, A., Pandey, C. M., Lalla, R. S., Garg, R. K., & Gupta, R. K. (2013). DTI correlates of cognition in term children with spastic diplegic cerebral palsy. European Journal of Paediatric Neurology, 17(3), 294–301. 10.1016/j.ejpn.2012.11.005 23246381

[IMAG.a.122-b95] Rothman, K. J. (1990). No adjustments are needed for multiple comparisons. Epidemiology (Cambridge, Mass.), 1(1), 43–46. 10.1097/00001648-199001000-00010 2081237

[IMAG.a.122-b96] Sheth, B. R., & Young, R. (2016). Two visual pathways in primates based on sampling of space: Exploitation and exploration of visual information. Frontiers in Integrative Neuroscience, 10, 37. 10.3389/fnint.2016.00037 27920670 PMC5118626

[IMAG.a.122-b97] Smith, R., Dhollander, T., & Connelly, A. (2019). On the regression of intracranial volume in Fixel-Based Analysis. In: Proc. Intl. Soc. Mag. Reson. Med. 27, Abstract #3385. https://archive.ismrm.org/2019/3385.html

[IMAG.a.122-b98] Stereo Optical Corporation. (2024). Stereo Fly Test. https://www.stereooptical.com/products/stereotests-color-tests/original-stereo-fly/#1529520225990-6a35365b-d00f

[IMAG.a.122-b99] Swienton, D. J., & Thomas, A. G. (2014). The visual pathway-functional anatomy and pathology. Seminars in Ultrasound, CT and MRI, 35(5), 487–503. 10.1053/j.sult.2014.06.007 25217301

[IMAG.a.122-b100] Takemura, H., Rokem, A., Winawer, J., Yeatman, J. D., Wandell, B. A., & Pestilli, F. (2016). A major human white matter pathway between dorsal and ventral visual cortex. Cerebral Cortex (New York, N.Y.: 1991), 26(5), 2205–2214. 10.1093/cercor/bhv064 25828567 PMC4830295

[IMAG.a.122-b101] Thiebaut de Schotten, M., Dell’Acqua, F., Forkel, S. J., Simmons, A., Vergani, F., Murphy, D. G. M., & Catani, M. (2011). A lateralized brain network for visuospatial attention. Nature Neuroscience, 14(10), 1245–1246. 10.1038/nn.2905 21926985

[IMAG.a.122-b102] Thompson, D. K., Thai, D., Kelly, C. E., Leemans, A., Tournier, J.-D., Kean, M. J., Lee, K. J., Inder, T. E., Doyle, L. W., Anderson, P. J., & Hunt, R. W. (2014). Alterations in the optic radiations of very preterm children—Perinatal predictors and relationships with visual outcomes. NeuroImage: Clinical, 4, 145–153. 10.1016/j.nicl.2013.11.007 24371797 PMC3871291

[IMAG.a.122-b103] Tomczak, M., & Tomczak, E. (2014). The need to report effect size estimates revisited. An overview of some recommended measures of effect size. Trends in Sport Sciences, 21(1), 77–84. 10.18038/estubtda.864226

[IMAG.a.122-b104] Tourbier, S., Aleman-Gomez, Y., Griffa, A., Bach Cuadra, M., & Hagmann, P. (2019). sebastientourbier/multiscalebrainparcellator: Multi-Scale Brain Parcellator v1.1.1 (Version v1.1.1) [Computer software]. Zenodo. 10.5281/zenodo.3627097

[IMAG.a.122-b105] Tournier, J.-D., Calamante, F., & Connelly, A. (2010). Improved probabilistic streamlines tractography by 2nd order integration over fibre orientation distributions. In: Proc. Intl. Soc. Mag. Reson. Med. 18, Abstract #1670. https://archive.ismrm.org/2010/1670.html

[IMAG.a.122-b106] Tournier, J.-D., Smith, R., Raffelt, D., Tabbara, R., Dhollander, T., Pietsch, M., Christiaens, D., Jeurissen, B., Yeh, C.-H., & Connelly, A. (2019). MRtrix3: A fast, flexible and open software framework for medical image processing and visualisation. NeuroImage, 202, 116137. 10.1016/j.neuroimage.2019.116137 31473352

[IMAG.a.122-b107] Tucker, J., & McGuire, W. (2004). Epidemiology of preterm birth. BMJ (Clinical Research Ed.), 329(7467), 675–678. 10.1136/bmj.329.7467.675 PMC517653 15374920

[IMAG.a.122-b108] Tustison, N. J., Avants, B. B., Cook, P. A., Yuanjie Zheng, Egan, A., Yushkevich, P. A., & Gee, J. C. (2010). N4ITK: Improved N3 bias correction. IEEE Transactions on Medical Imaging, 29(6), 1310–1320. 10.1109/TMI.2010.2046908 20378467 PMC3071855

[IMAG.a.122-b109] Veraart, J., Novikov, D. S., Christiaens, D., Ades-Aron, B., Sijbers, J., & Fieremans, E. (2016). Denoising of diffusion MRI using random matrix theory. NeuroImage, 142, 394–406. 10.1016/j.neuroimage.2016.08.016 27523449 PMC5159209

[IMAG.a.122-b110] Verly, M., Gerrits, R., Sleurs, C., Lagae, L., Sunaert, S., Zink, I., & Rommel, N. (2019). The mis-wired language network in children with developmental language disorder: Insights from DTI tractography. Brain Imaging and Behavior, 13(4), 973–984. 10.1007/s11682-018-9903-3 29934818

[IMAG.a.122-b111] Wilson, B., Cockburn, J., & Halligan, P. (1987). Development of a behavioral test of visuospatial neglect. Archives of Physical Medicine and Rehabilitation, 68(2), 98–102. https://pubmed.ncbi.nlm.nih.gov/3813864/3813864

[IMAG.a.122-b112] World Health Organization. (2014). Global nutrition targets 2025: Low birth weight policy brief. https://www.who.int/publications/i/item/WHO-NMH-NHD-14.5

