# Supplementary material for: Neural micro and macrostructural correlates of visual outcomes in children with unilateral cerebral palsy: A fixel-based study
Source: Imaging Neurosci (Camb). 2025 Aug 26;3:IMAG.a.122. doi: 10.1162/IMAG.a.122 (PMC12392306; doi:10.1162/IMAG.a.122)
Supplement: Supplementary Material [file IMAG.a.122_supp.pdf]

Supplementary material

Figure S1. Flow chart describing the study cohort

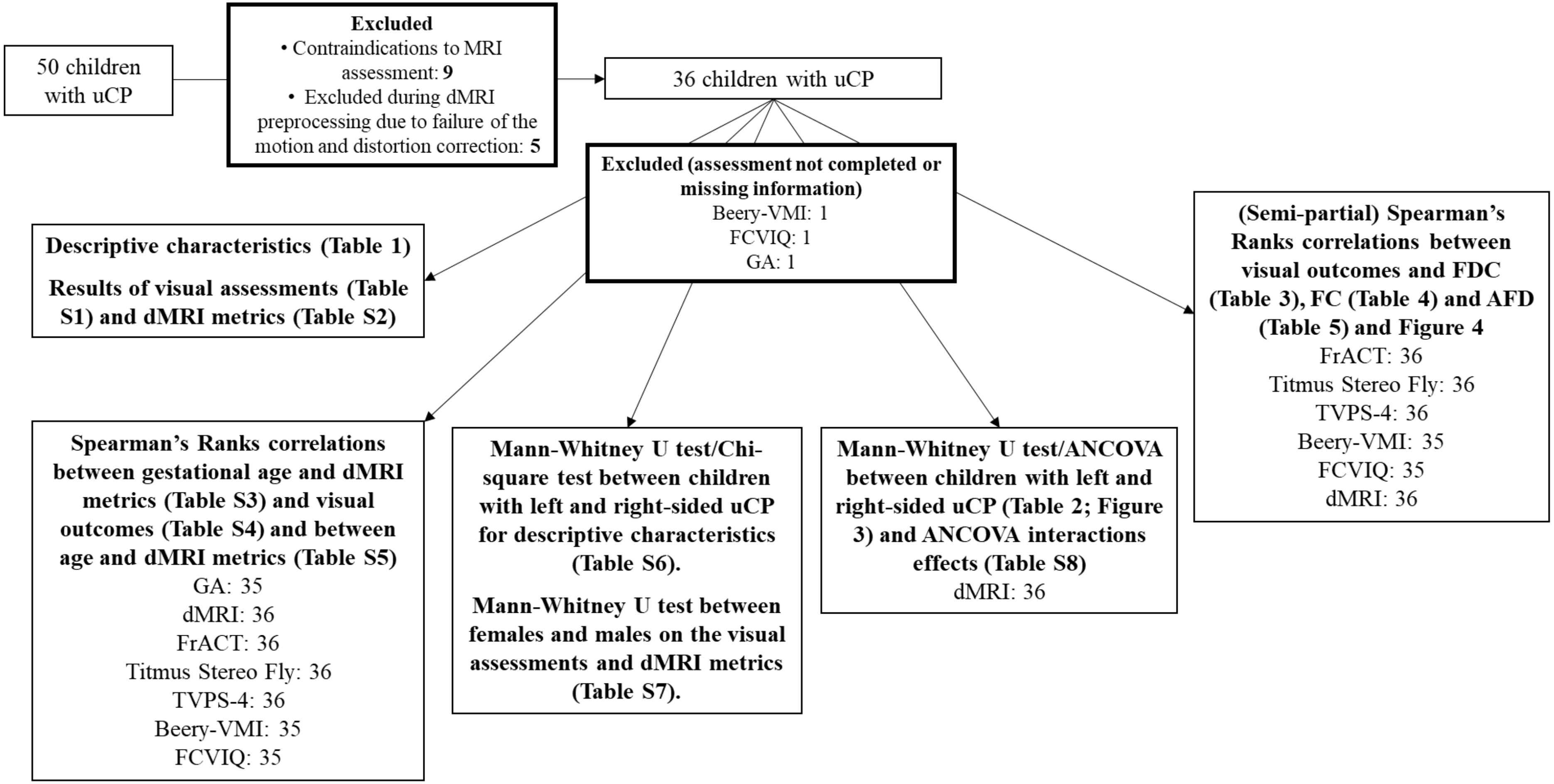

uCP, unilateral cerebral palsy; FrACT, Freiburg Visual Acuity Test; TVPS-4, Test of Visual Perceptual Skills, Fourth Edition; Beery-VMI, Beery-Buktenica Test of Visual-Motor Integration, Sixth Edition; FCVIQ, Flemish cerebral visual impairment questionnaire; GA, gestational age; dMRI, diffusion MRI; AFD, apparent fiber density; FC, fiber-bundle cross-section; FDC, fiber density and cross-section.

Figure S2. Percentage of cases presenting different lesion severity according to the semiquantitative MRI scores: a) global score (maximum score of 40); b) corpus callosum lesion distribution; c) lobar and subcortical score (including right and left side) ranging from 0 to 6.

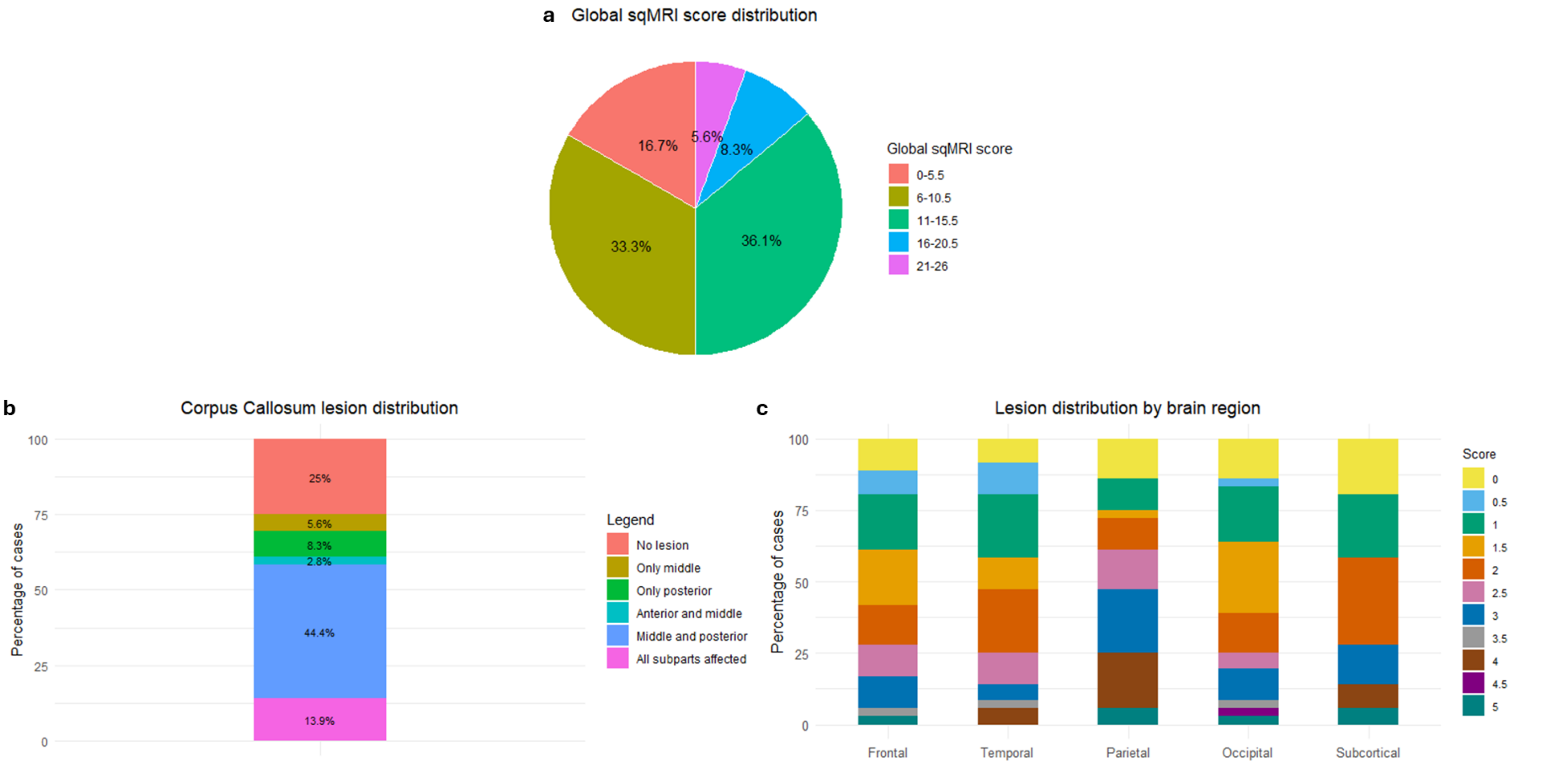

Table S1. Results of the visual assessments in children with unilateral cerebral palsy (*N*=36).

Visual assessments	Mean (SD), Median [IQR]	*N* (%) of children with impairments
[a]FrACT ↓	-0.12 [0.21]	3 (8)
[b]Titmus Stereo Fly ↑	9 [2.75]	9 (25)
[c]TVPS -Visual Discrimination ↑	-0.388 (1.132)	12 (33)
[c]TVPS -Spatial Relationships ↑	0 [1.67]	10 (28)
[c]TVPS -Form Constancy ↑	-0.222 (1.133)	9 (25)
[c]TVPS -Visual Figure-Ground ↑	-0.417 (1.137)	11 (31)
[c]TVPS -Visual Closure ↑	-0.593 (1.06)	15 (42)
[c]Beery- Visuomotor integration ↑	-1.07 [1.535]	20 (57)*
[d]FCVIQ ↓	4 [5]	Not applicable

[a]Results are reported in LogMAR. [b]Results report the last circle identified or the fly test. [c]Results are reported in *z*-scores. [d]Results calculated as the sum of the 'yes' items (1: the child presents the characteristic described in the item).[e]Results of the semiquantitative scale. ↑: higher values indicate a better performance. ↓: lower values indicate a better performance.
SD; standard deviation; IQR, interquartile range; *N*, Number of children; %**,** percentage of children out of the total sample of 36 children (* results calculated out of 35 due to NA data); FrACT, Freiburg Visual Acuity Test; TVPS, Test of Visual Perceptual Skills, Fourth Edition; Beery, Beery-Buktenica Test of Visual-Motor Integration, Sixth Edition; FCVIQ, Flemish cerebral visual impairment questionnaire.

Table S2. Results of the fixel metrics in children with unilateral cerebral palsy (*N*=36).

Visual tracts	↑ AFD Mean (SD), Median [IQR]	↑ FC Mean (SD), Median [IQR]	↑ FDC Mean (SD), Median [IQR]
Occipital CC	0.555 [0.064]	-0.042 (0.243)	0.533 (0.17)
Left IFOF	0.468 [0.037]	-0.04 (0.218)	0.453 (0.132)
Right IFOF	0.48 [0.045]	-0.018 (0.17)	0.479 (0.107)
Left ILF	0.455 [0.041]	0.031 (0.266)	0.483 (0.152)
Right ILF	0.456 [0.052]	0.04 (0.246)	0.485 (0.136)
Right OR	0.487 [0.046]	-0.05 (0.211)	0.470 (0.129)
Left OR	0.456 [0.044]	-0.043 (0.245)	0.451 (0.141)
Left SLF I	0.455 [0.042]	-0.037 (0.344)	0.451 (0.181)
Right SLF I	0.453 [0.072]	-0.036 (0.264)	0.442 (0.141)
Left SLF IId	0.417 [0.044]	-0.095 (0.301)	0.390 (0.166)
Right SLF IId	0.409 [0.044]	-0.096 (0.254)	0.386 (0.143)
Left SLF III	0.396 [0.034]	-0.098 (0.28)	0.368 (0.147)
Right SLF III	0.396 [0.034]	-0.088 (0.222)	0.373 (0.123)
Left SLF IIv	0.393 [0.044]	-0.085 (0.282)	0.368 (0.139)
Right SLF IIv	0.39 [0.031]	-0.083 (0.232)	0.369 (0.123)
Left VOF	0.417 [0.047]	0.056 (0.292)	0.458 (0.163)
Right VOF	0.456 (0.044)	0.04 (0.23)	0.497 (0.139)

SD; standard deviation; IQR, interquartile range; AFD, apparent fiber density; FC, fiber-bundle cross-section; FDC, fiber density and cross-section; CC, corpus callosum; IFOF, inferior fronto-occipital fasciculus; ILF, inferior longitudinal fasciculus; OR, optic radiation; SLF, superior longitudinal fasciculus; d, dorsal; v, ventral; VOF, vertical occipital fasciculus; ↑: lower values indicate axonal loss (AFD), reduction of bundle diameter (FC) or a combination of both (FDC).

Table S3. Spearman Ranks correlation between gestational age and the fixel metrics of the visual tracts.

	AFD			FC			FDC		
**Visual tracts**	r_s	***p*-value**	**FDR *p***	**r$_s$**	***p*-value**	**FDR *p***	r_s	***p*-value**	**FDR *p***
Occipital CC	0.130	0.455	0.848	0.242	0.161	0.663	0.270	0.117	0.545
Left IFOF	0.042	0.811	0.935	0.228	0.187	0.663	0.236	0.172	0.545
Right IFOF	0.032	0.854	0.935	0.144	0.410	0.663	0.136	0.437	0.575
Left ILF	0.194	0.264	0.848	0.217	0.210	0.663	0.230	0.184	0.545
Right ILF	0.106	0.546	0.848	0.102	0.558	0.678	0.135	0.440	0.575
Left OR	0.187	0.283	0.848	0.351	**0.039**	0.657	0.324	0.058	0.545
Right OR	0.139	0.427	0.848	0.259	0.133	0.663	0.233	0.178	0.545
Left SLF I	0.108	0.539	0.848	0.149	0.394	0.663	0.185	0.288	0.545
Right SLF I	-0.143	0.414	0.848	0.117	0.502	0.678	0.100	0.567	0.689
Left SLF IId	0.002	0.993	0.993	0.172	0.324	0.663	0.212	0.223	0.545
Right SLF IId	-0.179	0.303	0.848	0.062	0.722	0.767	0.002	0.990	0.990
Left SLF III	0.041	0.814	0.935	0.184	0.289	0.663	0.189	0.278	0.545
Right SLF III	-0.165	0.344	0.848	0.032	0.856	0.856	0.021	0.904	0.990
Left SLF IIv	0.026	0.880	0.935	0.174	0.316	0.663	0.204	0.240	0.545
Right SLF IIv	-0.106	0.546	0.848	0.065	0.711	0.767	-0.012	0.948	0.990
Left VOF	0.105	0.548	0.848	0.138	0.429	0.663	0.171	0.325	0.549
Right VOF	0.060	0.731	0.935	0.107	0.539	0.678	0.161	0.355	0.549

Significant results are shown in bold: $*p \leq 0.05$, $**p \leq 0.01$.
AFD, apparent fiber density; FC, fiber-bundle cross-section; FDC, fiber density and cross-section; CC, corpus callosum; IFOF, inferior fronto-occipital fasciculus; ILF, inferior longitudinal fasciculus; OR, optic radiation; SLF, superior longitudinal fasciculus; d, dorsal; v, ventral; VOF, vertical occipital fasciculus; r_s, Spearman correlations interpreted as no or negligible correlation (<0.30), low (0.30–0.49), moderate (0.50–0.69), high (0.70–0.89), or very high (≥0.90) (Mukaka, 2012); FDR, false discovery rate ($p \leq 0.05$).

Table S4. Spearman Ranks correlation between gestational age and the visual outcomes.

Visual outcomes	r_s	*p*-value	FDR *p*
Titmus Stereo Fly	0.156	0.371	0.645
FrACT	-0.421	**0.012**	0.106
TVPS-4 Visual Discrimination	0.138	0.430	0.645
TVPS-4 Spatial Relationships	0.061	0.730	0.938
TVPS-4 Form Constancy	0.279	0.104	0.291
TVPS-4 Visual Figure-Ground	0.009	0.958	0.958
TVPS-4 Visual Closure	0.282	0.101	0.291
Beery-Visuomotor integration	0.265	0.129	0.291
FCVIQ	0.032	0.860	0.958

Significant results are shown in bold: $*p \leq 0.05$, $**p \leq 0.01$.
FrACT, Freiburg Visual Acuity Test; TVPS-4, Test of Visual Perceptual Skills, Fourth Edition; Beery, Beery-Buktenica Test of Visual-Motor Integration, Sixth Edition; FCVIQ, Flemish cerebral visual impairment questionnaire; r_s, Spearman correlations interpreted as no or negligible correlation (<0.30), low (0.30–0.49), moderate (0.50–0.69), high (0.70–0.89), or very high (≥0.90) (Mukaka, 2012); FDR, false discovery rate ($p \leq 0.05$).

Table S5. Spearman Ranks correlation between age and the fixel metrics of the visual tracts.

	AFD			FC			FDC		
**Visual tracts**	r_s	***p*-value**	**FDR *p***	r_s	***p*-value**	**FDR *p***	r_s	***p*-value**	**FDR *p***
Occipital CC	0.016	0.925	0.925	**0.506****	**0.002**	**0.007**	**0.437***	**0.008**	**0.021**
Left IFOF	-0.038	0.828	0.879	**0.442***	**0.007**	**0.012**	**0.379***	**0.023**	**0.039**
Right IFOF	-0.234	0.170	0.426	0.299	0.076	0.092	0.235	0.167	0.178
Left ILF	-0.212	0.215	0.426	**0.519****	**0.001**	**0.007**	**0.449***	**0.006**	**0.021**
Right ILF	-0.223	0.192	0.426	**0.438***	**0.007**	**0.012**	0.329	0.050	0.064
Left OR	-0.049	0.777	0.879	**0.441***	**0.007**	**0.012**	**0.427***	**0.009**	**0.021**
Right OR	-0.195	0.253	0.426	0.288	0.089	0.094	0.207	0.226	0.226
Left SLF I	0.051	0.768	0.879	**0.519****	**0.001**	**0.007**	**0.424***	**0.010**	**0.021**
Right SLF I	0.218	0.201	0.426	0.258	0.128	0.128	0.309	0.067	0.076
Left SLF IId	0.254	0.135	0.426	**0.494****	**0.002**	**0.007**	**0.446***	**0.006**	**0.021**
Right SLF IId	0.223	0.192	0.426	0.294	0.081	0.092	0.325	0.053	0.064
Left SLF III	0.191	0.264	0.426	**0.466***	**0.004**	**0.011**	**0.462***	**0.005**	**0.021**
Right SLF III	0.342	0.041	0.426	**0.354***	**0.034**	**0.045**	**0.363***	**0.030**	**0.046**
Left SLF IIv	0.134	0.435	0.569	**0.464***	**0.004**	**0.011**	**0.443***	**0.007**	**0.021**
Right SLF IIv	0.308	0.068	0.426	**0.370***	**0.026**	**0.037**	**0.388***	**0.019**	**0.037**
Left VOF	-0.143	0.407	0.569	**0.610****	**<0.001**	**0.001**	**0.455***	**0.005**	**0.021**
Right VOF	-0.187	0.276	0.426	**0.453***	**0.006**	**0.012**	0.330	0.049	0.064

Significant results are shown in bold: **p* FDR ≤ 0.05, ***p* FDR ≤ 0.01.

AFD, apparent fiber density; FC, fiber-bundle cross-section; FDC, fiber density and cross-section; CC, corpus callosum; IFOF, inferior fronto-occipital fasciculus; ILF, inferior longitudinal fasciculus; OR, optic radiation; SLF, superior longitudinal fasciculus; d, dorsal; v, ventral; VOF, vertical occipital fasciculus. r_s, Spearman correlations interpreted as no or negligible correlation (<0.30), low (0.30–0.49), moderate (0.50–0.69), high (0.70–0.89), or very high (≥0.90) (Mukaka, 2012); FDR, false discovery rate ($p \leq 0.05$).

Table S6. Descriptive characteristics of children with left and right-side uCP.

	**Children with left-sided uCP (*N*=17)**	**Children with right-sided uCP (*N*=19)**	***p*-value**
Age, median [IQR] in years:months	10:08 [05:01]	11:06 [05:02]	[a]0.99
Gestational age, median [IQR] in months	38 [4]	37 [6.5]	[a]0.63
Intracranial brain volume, median [IQR] in cm^3	1378.063 [79.432]	1389.934 [77.383]	[a]0.78
Sex: Male (n, %)	11 (31)	8 (22)	[b]0.31

[a] Results of the Mann-Whitney U-tests. [b] Results of the Chi-square tests.
N, number of children; IQR, interquartile range. *$p \leq 0.05$.

Table S7. Differences between females and males in children with uCP on the visual outcomes and the fixel metrics of the visual tracts.

Visual outcomes	Males<br>Median [IQR]<br>*N*=19	Female<br>Median [IQR]<br>*N*=17	*p*-value	FDR *p*
FrACT	-0.12 [0.375]	-0.12 [0.18]	0.590	0.960
Titmus Stereo Fly	8 [6.5]	9 [1]	0.249	0.960
TVPS-4 Visual Discrimination	-0.33 [2]	-0.33 [0.67]	0.911	0.985
TVPS-4 Spatial Relationships	0 [1.835]	0.67 [1]	0.343	0.960
TVPS-4 Form Constancy	-0.33 [1.335]	-0.33 [1.34]	0.544	0.960
TVPS-4 Visual Figure-Ground	-0.33 [1.83]	-0.33 [1.34]	0.381	0.960
TVPS-4 Visual Closure	-0.67 [1.17]	-0.33 [1.66]	0.296	0.960
Beery-Visuomotor integration	-2.1 [2.155]	-0.87 [0.54]	**0.015***	0.420
FCVIQ	6 [6.5]	2.5 [4.25]	**0.039***	0.546
**Visual tracts**				
Occipital CC	0.517 [0.126]	0.486 [0.299]	0.531	0.960
Left IFOF	0.456 [0.147]	0.468 [0.197]	0.573	0.960
Right IFOF	0.480 [0.100]	0.482 [0.147]	0.754	0.960
Left ILF	0.503 [0.179]	0.496 [0.236]	0.300	0.960
Right ILF	0.466 [0.167]	0.450 [0.249]	0.925	0.990
Left OR	0.461 [0.197]	0.481 [0.252]	0.397	0.960
Right OR	0.462 [0.174]	0.476 [0.196]	0.661	0.960
Left SLF I	0.466 [0.203]	0.398 [0.267]	0.433	0.960
Right SLF I	0.467 [0.235]	0.433 [0.101]	0.731	0.960
Left SLF IId	0.403 [0.182]	0.408 [0.265]	0.594	0.960
Right SLF IId	0.357 [0.151]	0.377 [0.110]	0.950	0.990
Left SLF III	0.356 [0.172]	0.402 [0.235]	0.661	0.960
Right SLF III	0.365 [0.139]	0.363 [0.129]	1.000	1.000
Left SLF IIv	0.376 [0.155]	0.399 [0.218]	0.684	0.960
Right SLF IIv	0.350 [0.122]	0.355 [0.145]	0.900	0.990
Left VOF	0.450 [0.154]	0.488 [0.299]	0.707	0.960
Right VOF	0.497 [0.146]	0.507 [0.216]	0.731	0.960

Significant results are shown in bold: *$p \leq 0.05$.
N, number of children; IQR, interquartile range; FrACT, Freiburg Visual Acuity Test; TVPS, Test of Visual Perceptual Skills, Fourth Edition; Beery, Beery-Buktenica Test of Visual-Motor Integration, Sixth Edition; FCVIQ, Flemish cerebral visual impairment questionnaire; AFD, apparent fiber density; FC, fiber-bundle cross-section; FDC, fiber density and cross-section; CC, corpus callosum; IFOF, inferior fronto-occipital fasciculus; ILF, inferior longitudinal fasciculus; OR, optic radiation; SLF, superior longitudinal fasciculus; d, dorsal; v, ventral; VOF, vertical occipital fasciculus.

Table S8. Interaction effect of the ANCOVA analysis between children with left and right-sided uCP.

	Fiber cross-section (FC)						Fiber density and cross-section (FDC)					
	Interaction Group*Age			Interaction Group *ICV			Interaction Group*Age			Interaction group*ICV		
**Visual tracts**	***F***	***p***	η_p^2	***F***	***p***	η_p^2	***F***	***p***	η_p^2	***F***	***p***	η_p^2
Occipital CC	3.696	0.064	0.11	0.121	0.73	0.004	2.044	0.163	0.064	0.39	0.537	0.013
Left IFOF	0.767	0.388	0.025	0.765	0.389	0.025	0.631	0.433	0.021	2.429	0.13	0.075
Right IFOF	2.911	0.098	0.088	0.042	0.838	0.001	2.017	0.166	0.063	0.345	0.561	0.011
Left ILF	0.824	0.371	0.027	0.258	0.615	0.009	0.559	0.46	0.018	1.315	0.261	0.042
Right ILF	2.555	0.12	0.078	0.227	0.637	0.008	1.562	0.221	0.049	0.123	0.728	0.004
Left OR	1.116	0.299	0.036	0.98	0.33	0.032	0.328	0.571	0.011	2.923	0.098	0.089
Right OR	3.632	0.066	0.108	0.278	0.602	0.009	2.767	0.107	0.084	0.000	0.996	0.000
Left SLF I	2.219	0.147	0.069	0.314	0.579	0.01	0.102	0.751	0.003	1.768	0.194	0.056
Right SLF I	1.332	0.258	0.043	0.534	0.47	0.018	1.076	0.308	0.035	0.027	0.871	0.001
Left SLF IId	0.237	0.63	0.008	0.79	0.381	0.026	0.008	0.931	0.000	1.1	0.303	0.035
Right SLF IId	2.234	0.145	0.069	0.282	0.599	0.009	2.21	0.148	0.069	0.102	0.752	0.003
Left SLF III	0.092	0.764	0.003	0.676	0.417	0.022	0.009	0.924	0.000	1.183	0.285	0.038
Right SLF III	2.024	0.165	0.063	0.105	0.748	0.003	1.742	0.197	0.055	0.298	0.589	0.01
Left SLF IIv	0.094	0.762	0.003	1.293	0.264	0.041	0.019	0.89	0.001	1.652	0.209	0.052
Right SLF IIv	1.648	0.209	0.052	0.439	0.513	0.014	1.997	0.168	0.062	0.198	0.659	0.007
Left VOF	1.342	0.256	0.043	0.043	0.837	0.001	0.111	0.741	0.004	0.04	0.843	0.001
Right VOF	**6.473**	**0.016***	**0.177**	2.093	0.158	0.065	**4.525**	**0.042***	**0.131**	0.423	0.52	0.014

Significant results are shown in bold: *$p \leq 0.05$, **$p \leq 0.01$.

FC, fiber-bundle cross-section; FDC, fiber density and cross-section; ICV; intracranial volume; F, F-tests value; p; p-value; η_p^2; effect sizes calculated using partial η squared and interpreted as small (0.01–0.06), medium (0.06–0.14), or large (>0.14) (Hinkle et al., 2003). CC, corpus callosum; IFOF, inferior fronto-occipital fasciculus; ILF, inferior longitudinal fasciculus; OR, optic radiation; SLF, superior longitudinal fasciculus; d, dorsal; v, ventral; VOF, vertical occipital fasciculus.

Table S9. Differences in fiber-bundle cross-section (FC) and fiber density and cross-section (FDC) of the visual tracts between children with left and right-sided that remained statistically significant after false discovery rate (FDR) correction.

	**ANCOVA corrected for age and ICV on FC**		**ANCOVA corrected for age and ICV on FDC**	
**Visual tracts**	**Main effect group**		**Main effect group**	
	p	**FDR p**	***p***	**FDR p**
Occipital CC	0.38	0.462	0.244	0.296
Left IFOF	0.334	0.437	0.242	0.296
Right IFOF	**0.009**	**0.029**	**0.029**	0.070
Left ILF	0.517	0.549	0.401	0.426
Right ILF	0.073	0.155	0.074	0.126
Left OR	0.629	0.629	0.546	0.546
Right OR	**0.011**	**0.029**	**0.018**	0.052
Left SLF I	**0.012**	**0.029**	**0.01**	**0.032**
Right SLF I	**0.012**	**0.029**	**0.001**	**0.006**
Left SLF IId	0.149	0.266	0.074	0.126
Right SLF IId	**0.002**	**0.016**	**<0.001**	**0.006**
Left SLF III	0.200	0.284	0.057	0.121
Right SLF III	**0.001**	**0.014**	**0.001**	**0.006**
Left SLF IIv	0.172	0.266	0.087	0.135
Right SLF IIv	**0.003**	**0.016**	**0.002**	**0.010**
Left VOF	0.495	0.549	0.396	0.426
Right VOF	0.151	0.266	0.118	0.180

Significant results are shown in bold: *$p \leq 0.05$, **$p \leq 0.01$. FDR, false discovery rate ($p \leq 0.05$).
FC, fiber-bundle cross-section; FDC, fiber density and cross-section; ICV; intracranial volume; F, F-tests value; p; p-value; CC, corpus callosum; IFOF, inferior fronto-occipital fasciculus; ILF, inferior longitudinal fasciculus; OR, optic radiation; SLF, superior longitudinal fasciculus; d, dorsal; v, ventral; VOF, vertical occipital fasciculus.

Table S10. Spearman Rank's correlations between the results of the visual assessments and the AFD of the visual tracts after FDR correction.

		Apparent fiber density (AFD)																
**Visual assessments**		**Occipital CC**	**Left IFOF**	**Right IFOF**	**Left ILF**	**Right ILF**	**Left OR**	**Right OR**	**Left SLF I**	**Right SLF I**	**Left SLF IId**	**Right SLF IId**	**Left SLF III**	**Right SLF III**	**Left SLF IIv**	**Right SLF IIv**	**Left VOF**	**Right VOF**
**FraCT**	r_s	**-0.518***	-0.393	**-0.402***	**-0.513***	**-0.423***	**-0.448***	**-0.557***	-0.229	**-0.412***	-0.386	**-0.406***	-0.304	-0.345	**-0.416***	-0.306	**-0.412***	**-0.534***
	p	0.016	0.051	0.048	0.016	0.041	0.032	0.012	0.222	0.045	0.055	0.047	0.117	0.081	0.045	0.117	0.045	0.012
**Titmus Stereo Fly**	r_s	0.324	**0.452***	**0.543***	0.385	0.381	0.336	**0.461***	0.251	0.350	0.317	0.287	0.380	0.193	**0.432***	0.246	0.331	**0.571***
	p	0.098	0.031	0.012	0.055	0.057	0.09	0.028	0.192	0.078	0.106	0.14	0.057	0.304	0.038	0.195	0.091	0.012
**TVPS-4 Visual Discrimination**	r_s	0.253	0.329	0.285	0.347	**0.443***	0.354	0.336	0.133	0.260	0.171	0.266	0.100	0.179	0.136	0.100	0.128	0.317
	p	0.191	0.092	0.14	0.08	0.034	0.077	0.09	0.469	0.18	0.362	0.174	0.577	0.341	0.461	0.577	0.484	0.106
**TVPS-4 Spatial Relationships**	r_s	0.335	0.370	0.345	0.377	**0.541***	**0.406***	**0.422***	0.139	0.136	0.195	0.209	0.263	0.163	0.243	0.116	0.258	**0.497***
	p	0.090	0.063	0.081	0.059	0.012	0.047	0.041	0.461	0.461	0.302	0.268	0.176	0.379	0.2	0.528	0.183	0.018
**TVPS-4 Form Constancy**	r_s	**0.503***	0.329	0.376	0.391	**0.466***	**0.408***	**0.488***	0.169	0.332	0.250	0.261	0.226	0.230	0.240	0.107	0.185	**0.549***
	p	0.018	0.092	0.059	0.052	0.028	0.047	0.020	0.363	0.091	0.193	0.179	0.228	0.222	0.203	0.56	0.326	0.012
**TVPS-4 Visual Figure-Ground**	r_s	0.369	**0.428***	0.395	0.388	**0.540***	**0.447***	**0.508***	0.137	0.310	0.263	0.347	0.247	0.293	0.288	0.283	0.193	0.360
	p	0.063	0.039	0.051	0.054	0.012	0.032	0.017	0.461	0.113	0.176	0.08	0.194	0.135	0.14	0.144	0.304	0.071
**TVPS-4 Visual Closure**	r_s	**0.435***	0.249	0.305	0.358	**0.461***	**0.460***	**0.432***	0.236	0.212	0.196	0.314	0.248	0.333	0.287	0.255	0.308	**0.398***
	p	0.038	0.194	0.117	0.073	0.028	0.028	0.038	0.211	0.264	0.300	0.110	0.194	0.091	0.14	0.187	0.114	0.050
**Beery- Visuomotor integration**	r_s	0.291	0.291	0.358	**0.421***	**0.463***	**0.403***	**0.418***	0.178	-0.106	0.080	0.011	0.210	-0.070	0.245	-0.016	0.400	**0.575***
	p	0.140	0.140	0.077	0.045	0.029	0.05	0.045	0.349	0.567	0.66	0.949	0.272	0.699	0.202	0.934	0.051	0.012
**FCVIQ**	r_s	**-0.521***	**-0.410***	**-0.558***	**-0.507***	**-0.486***	**-0.468***	**-0.541***	-0.245	-0.357	-0.166	**-0.499***	-0.278	-0.376	-0.317	-0.382	**-0.439***	**-0.502***
	p	0.016	0.047	0.012	0.018	0.022	0.028	0.012	0.202	0.077	0.379	0.018	0.159	0.063	0.11	0.059	0.038	0.018

Cases were excluded pairwise. Significant results are shown in bold: $*p \leq 0.05$, $**p \leq 0.01$.
CC, corpus callosum; IFOF, inferior fronto-occipital fasciculus; ILF, inferior longitudinal fasciculus; OR, optic radiation; SLF, superior longitudinal fasciculus; d, dorsal; v, ventral; VOF, vertical occipital fasciculus; FrACT, Freiburg Visual Acuity Test; TVPS-4, Test of Visual Perceptual Skills, Fourth Edition; Beery, Beery-Buktenica Test of Visual-Motor Integration, Sixth Edition; FCVIQ, Flemish cerebral visual impairment questionnaire. r_s, Spearman correlations interpreted as no or negligible correlation (<0.30), low (0.30–0.49), moderate (0.50–0.69), high (0.70–0.89), or very high (≥0.90) (Mukaka, 2012); *p*, p-value after false discovery rate (FDR) correction.
